# Posterior Basolateral Amygdala is a Critical Amygdaloid Area for Temporal Lobe Epilepsy

**DOI:** 10.1002/advs.202407525

**Published:** 2024-10-30

**Authors:** Yan‐Hui Sun, Bo‐Wu Hu, Li‐Heng Tan, Lin Lin, Shu‐Xia Cao, Tan‐Xia Wu, Hao Wang, Bin Yu, Qin Wang, Hong Lian, Jiadong Chen, Xiao‐Ming Li

**Affiliations:** ^1^ Department of Neurology and Department of Psychiatry of the Second Affiliated Hospital School of Medicine Zhejiang University Hangzhou 310058 China; ^2^ NHC and CAMS Key Laboratory of Medical Neurobiology Ministry of Education Frontier Science Center for Brain Research and Brain‐Machine Integration School of Brain Science and Brain Medicine Zhejiang University Hangzhou 310058 China; ^3^ Department of Neurobiology of Sir Run Shaw Hospital Zhejiang University Hangzhou 310058 China; ^4^ Nanhu Brain‐computer Interface Institute Hangzhou 311100 China; ^5^ Affiliated Mental Health Center and Hangzhou Seventh People's Hospital Zhejiang University Hangzhou 310013 China; ^6^ Key Laboratory of Novel Targets Drug Study for Neural Repair of Zhejiang Province School of Medicine Hangzhou City University Hangzhou 310015 China; ^7^ Research Center of System Medicine School of Basic Medical Sciences Zhejiang University Hangzhou 310058 China

**Keywords:** feedback inhibition, neural circuits, posterior basolateral amygdala, temporal lobe epilepsy

## Abstract

The amygdaloid complex consists of multiple nuclei and is a key node in controlling temporal lobe epilepsy (TLE) in both human and animal model studies. However, the specific nucleus in the amygdaloid complex and the neural circuitry governing seizures remain unknown. Here, it is discovered that activation of glutamatergic neurons in the posterior basolateral amygdala (pBLA) induces severe seizures and even mortality. The pBLA glutamatergic neurons project collateral connections to multiple brain regions, including the insular cortex (IC), bed nucleus of the stria terminalis (BNST), and central amygdala (CeA). Stimulation of pBLA‐targeted IC neurons triggers seizures, whereas ablation of IC neurons suppresses seizures induced by activating pBLA glutamatergic neurons. GABAergic neurons in the BNST and CeA establish feedback inhibition on pBLA glutamatergic neurons. Deleting GABAergic neurons in the BNST or CeA leads to sporadic seizures, highlighting their role in balancing pBLA activity. Furthermore, pBLA neurons receive glutamatergic inputs from the ventral hippocampal CA1 (vCA1). Ablation of pBLA glutamatergic neurons mitigates both acute and chronic seizures in the intrahippocampal kainic acid‐induced mouse model of TLE. Together, these findings identify the pBLA as a pivotal nucleus in the amygdaloid complex for regulating epileptic seizures in TLE.

## Graphical abstract

This study identifies the posterior basolateral amygdala (pBLA) as a critical nucleus in the regulation of temporal lobe epilepsy (TLE) within the amygdaloid complex. It highlights the distinct roles of both upstream and downstream neural circuits of the pBLA in modulating epileptic seizures. These findings may facilitate more precise and effective interventions targeting neural circuits to treat TLE.

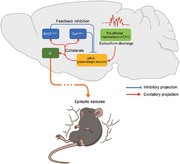

## 1 Introduction

Epilepsy, a prevalent neurological disorder, impacts an estimated 65 million individuals worldwide.^[^ ^1^ ^]^ It is characterized by the recurrent emergence of spontaneous seizures, primarily driven by heightened neuronal activity within the brain.^[^ ^2^ ^]^ Among the diverse types of epilepsy, temporal lobe epilepsy (TLE), originating within one or more anatomical regions of the temporal lobe,^[^ ^3^ ^]^ represents the most common and drug‐resistant form of epilepsy in adults.^[^ ^4^, ^5^, ^6^ ^]^

While various temporal lobe structures are implicated in TLE, the hippocampus has been the focus of most studies.^[^ ^7^, ^8^, ^9^, ^10^, ^11^ ^]^ In contrast, the amygdaloid complex has been less thoroughly investigated. Nevertheless, evidence from human and animal studies of TLE indicates that the amygdaloid complex plays a significant role in seizure generation, seizure activity, and epileptogenesis.^[^ ^12^, ^13^, ^14^ ^]^ Notably, a significant subpopulation of patients with TLE exhibit pronounced neuropathological changes in the amygdala, in addition to hippocampal damage.^[^ ^15^, ^16^ ^]^ Furthermore, magnetic resonance imaging (MRI) studies frequently reveal amygdalar volume reductions ranging from 10% to 30% in patients with TLE.^[^ ^17^ ^]^ Electrophysiological recordings in animal models and TLE patients have identified interictal activity within the amygdala.^[^ ^13^, ^18^ ^]^ Moreover, surgical resection of the temporal lobe in TLE patients can result in full seizure control only when the amygdala is also removed.^[^ ^19^ ^]^ In some cases, removal of the amygdala alone (amygdalectomy) is sufficient for seizure elimination.^[^ ^20^, ^21^ ^]^ The amygdaloid complex is structurally diverse, comprising over 10 nuclei,^[^ ^22^, ^23^, ^24^ ^]^ but the precise nucleus and circuitry responsible for epileptic seizures remain unidentified.

In this study, we elucidated the neural circuits of the posterior basolateral amygdala (pBLA) glutamatergic neurons in seizure genesis and propagation and identified pBLA as a key nucleus in the amygdaloid complex that regulates epileptic seizures in a mouse model of TLE.

## 2 Results

### 2.1 Optogenetic Activation of Glutamatergic Neurons in the pBLA Induces Epileptic Seizures and Even Death

**Figure 1:**
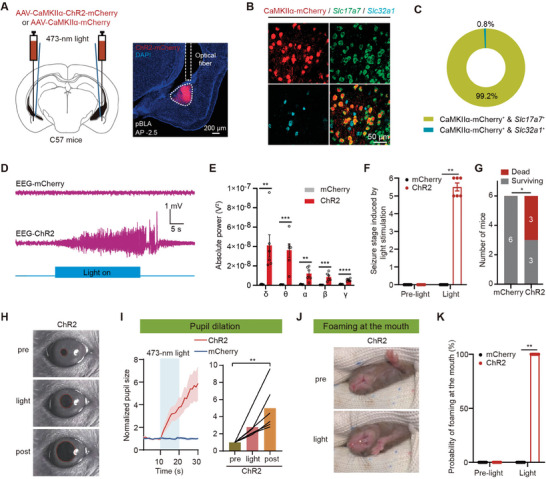
Optogenetic activation of pBLA glutamatergic neurons induces epileptic symptoms and even death. A) Left: Schematic of optogenetic activation of pBLA glutamatergic neurons; Right: Expression of ChR2‐mCherry in the pBLA region and the location of optical fiber. Anterior‐posterior axis (AP). B) Representative images of CaMKIIα‐mCherry‐labeled neurons in the pBLA co‐localized with *Slc17a7* / *Slc32a1*. C) Statistical graph of CaMKIIα‐mCherry‐labeled neurons in the pBLA co‐localized with *Slc17a7* / *Slc32a1* (*n* = 3 mice, 2 brain slices per mouse). D) Representative EEG trace of epileptiform discharges upon optogenetic activation of pBLA glutamatergic neurons. E) EEG spectral analysis of the epileptiform discharges induced by optogenetic activation of pBLA glutamatergic neurons (*n* = 6 mice per group; two‐tailed unpaired t‐test, **P < 0.01, ***P < 0.001, ****P < 0.0001). F) Seizure stage induced by optogenetic activation of pBLA glutamatergic neurons (*n* = 6 mice per group; Mann‐Whitney U test, **P < 0.01). G) Number of dead and surviving mice following optogenetic activation of pBLA glutamatergic neurons (Chi‐square test, *P < 0.05). H and I) Representative images and normalized curves of pupil changes induced by optogenetic activation of pBLA glutamatergic neurons (*n* = 6 mice per group; Friedman test followed by Dunn's multiple comparisons test, **P < 0.01). J and K) Images and probability of foaming at the mouth induced by optogenetic activation of pBLA glutamatergic neurons (*n* = 6 mice per group; Mann‐Whitney U test, **P < 0.01). Data are presented as mean ± standard error of the mean (SEM). AAV, adeno‐associated virus; AP, anterior‐posterior axis; CaMKIIα, calcium/calmodulin‐dependent protein kinase IIα; ChR2, channelrhodopsin‐2; DAPI, 4′,6‐diamidino‐2‐phenylindole; EEG, electroencephalography; pBLA, posterior basolateral amygdala.

As above mentioned, the specific nuclei within the amygdala that mediate seizures remain unclear. To manipulate glutamatergic neurons of the pBLA in C57BL/6J mice, we utilized adeno‐associated virus (AAV) tools controlled by the widely used calcium/calmodulin‐dependent protein kinase IIα (CaMKIIα) promoter. We injected AAV expressing channelrhodopsin‐2 (AAV‐CaMKIIα‐ChR2‐mCherry) or AAV‐CaMKIIα‐mCherry into the pBLA in C57BL/6J mice and bilaterally implanted optical fibers to optogenetically stimulate glutamatergic neurons in the pBLA (**Figure** **1**A). Three weeks after viral injection, the reliability of AAV tools controlled by the CaMKIIα promoter for infecting glutamatergic neurons in the pBLA was validated using fluorescence in situ hybridization (FISH). We found that 99.2% of virus‐infected pBLA neurons (CaMKIIα‐mCherry^+^) co‐localized with the glutamatergic neuron marker *Slc17a7* (encoding vesicular glutamate transporter 1 (VGluT1)) but had little co‐localization with the GABAergic inhibitory neuron marker *Slc32a1* (encoding vesicular GABA transporter (VGAT)) (Figure 1B,C).

Electroencephalography (EEG) recordings revealed that optogenetic activation of pBLA glutamatergic neurons induced pronounced epileptiform discharges lasting 39.75 ± 5.31 seconds (*n* = 6 mice) (Figure 1D), significantly increasing the power of five different frequency bands of brain waves: δ waves (0.5—4 Hz), θ waves (4—8 Hz), α waves (8—12 Hz), β waves (12—30 Hz), and γ waves (30—500 Hz) (Figure 1E). This activation also led to grade 5–6 seizures (Figure 1F), and elicited other seizure symptoms, including pupillary dilation and foaming at the mouth (Figure 1H–K). Remarkably, a brief 30‐second activation of pBLA glutamatergic neurons may result in sudden death of the animals (seizure stage 6; three out of six mice) (Figure 1F,G; Movie S1, Supporting Information). In contrast, optogenetic activation of glutamatergic neurons in the anterior BLA (aBLA) did not induce seizures (Figure S1, Supporting Information). These results underscore the pivotal role of hyperactive pBLA glutamatergic neurons in initiating epileptic seizures and severe seizure‐related death.

### 2.2 pBLA Glutamatergic Neurons Collaterally Project to Multiple Brain Regions

To explore the downstream brain regions of the pBLA that mediate epileptic seizures, we injected AAV‐CaMKIIα‐mCherry into the pBLA and observed mCherry‐labeled axon terminals in several brain regions, including the medial prefrontal cortex (mPFC), insular cortex (IC), bed nucleus of the stria terminalis (BNST), central amygdala (CeA), and ventromedial hypothalamus shell (VMH shell) (Figure S2, Supporting Information).

**Figure 2:**
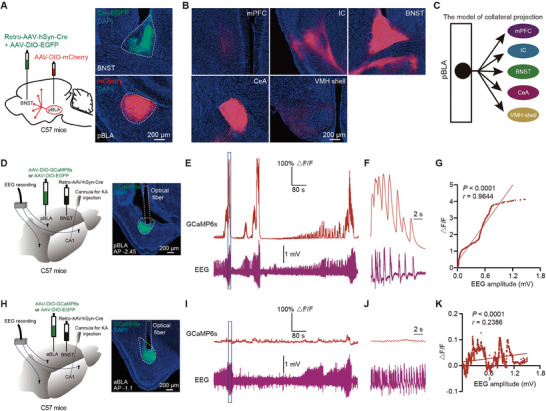
Calcium signals of the collateral‐projecting pBLA neurons are synchronous with seizure activity in the intrahippocampal KA‐induced acute seizure model. A) Left: Schematic diagram showing the strategy to retrogradely label pBLA projecting neurons with AAV (*n* = 3 mice). Right: Representative images showing injection sites in BNST and pBLA. B) Collateral projections of pBLA neurons retrogradely labeled from BNST in A). C) Model illustrating pBLA efferents as collateral projections. D and H) Left: Schematic of tracing strategy to identify BNST‐projecting pBLA or aBLA neurons and EEG recordings in the intrahippocampal KA‐induced acute seizure model (*n* = 5 mice per group). Right: Representative GCaMP6s expression and the location of optical fibers in pBLA or aBLA. E and I) Representative calcium signals of pBLA or aBLA neurons and EEG signals recorded in the intrahippocampal KA‐induced acute seizure model. F and J) Enlarged view of calcium signals and EEG signals from the blue boxes in E) and I). G and K) Linear regression analysis and Pearson's correlation of pBLA or aBLA neuronal activity and EEG signals from the blue boxes in E) and I) (two‐tailed Pearson's correlation, each point = 20 ms, with a total of 1000 points). AAV, adeno‐associated virus; AP, anterior‐posterior axis; DAPI, 4′,6‐diamidino‐2‐phenylindole; EEG, electroencephalography; EGFP, enhanced green fluorescent protein; KA, kainic acid; pBLA, posterior basolateral amygdala; aBLA, anterior basolateral amygdala; mPFC, medial prefrontal cortex; IC, insular cortex; BNST, bed nucleus of the stria terminalis; CeA, central amygdala; VMH shell, ventromedial hypothalamus shell.

Epileptic seizures are characterized by sudden and transient signs resulting from excessive and synchronous neuronal activity in the brain. Therefore, it is plausible that pBLA glutamatergic neurons may collaterally project to different brain regions, mediating this synchronous neuronal activity. To investigate the potential collateral projection pattern, we employed an intersectional strategy. Since the BNST emerged as a significant target region of the pBLA (Figure S2B,C, Supporting Information), we administered a combination of retrograde (Retro)‐AAV‐hSyn‐Cre and AAV‐DIO‐EGFP (enhanced green fluorescent protein) into the BNST and AAV‐DIO‐mCherry into the pBLA (**Figure** **2**A). Intriguingly, pBLA→BNST neurons (pBLA neurons retrogradely labeled from the BNST) exhibited collateral projections to the mPFC, IC, BNST, CeA, and VMH shell (Figure 2B). Furthermore, we conducted dual retrograde labeling experiments within the same mice, tracing back from distinct downstream targets: BNST and CeA, IC and BNST, and IC and CeA (Figure S3A,D,G, Supporting Information). These experiments revealed significant overlap in pBLA labeled neurons, with 69% of neurons double‐labeled from the BNST and CeA (Figure S3B,C, Supporting Information), 60% from the BNST and IC (Figure S3E,F, Supporting Information), and 63% from the IC and CeA (Figure S3H,I, Supporting Information). Overall, these findings substantiate the existence of significant collateral projections from pBLA glutamatergic neurons to multiple brain areas (Figure 2C).

### 2.3 The Activity of Collateral‐Projecting pBLA Neurons Synchronizes with EEG Signals

To study the activity of collateral‐projecting pBLA neurons during seizures, we specifically expressed the Ca^2+^ indicator GCaMP6s in pBLA neurons in a projection‐specific manner. The above results demonstrated that more than 60% of pBLA neurons traced from the BNST, CeA, or IC were co‐labeled (Figure S3C,F,I, Supporting Information). Additionally, a higher number of neurons were labeled from the BNST compared to the IC and CeA (Figure S3C,F,I, Supporting Information). Using FISH staining, we found that 100% of pBLA→BNST neurons co‐localized with *Slc17a7* (encoding VGluT1, a marker for glutamatergic neurons) but not with *Slc32a1* (encoding VGAT, a marker for GABAergic neurons) (Figure S3J–M, Supporting Information), indicating that pBLA→BNST neurons are exclusively glutamatergic, not GABAergic. Moreover, these pBLA→BNST neurons represent ≈75% of the total glutamatergic population within the pBLA (Figure S3K, Supporting Information).

Based on these findings, we chose to inject Retro‐AAV‐hSyn‐Cre into the BNST and AAV‐DIO‐GCaMP6s into the pBLA to track calcium activity in these neurons in a projection‐targeted manner (Figure 2D). Then, using an in vivo fiber photometry system, we monitored the calcium activity of collateral‐projecting pBLA neurons in the intrahippocampal kainic acid (KA)‐induced acute seizure model. By aligning calcium signals with EEG signals, we observed that the calcium activity of collateral‐projecting pBLA neurons synchronized with the EEG signals and showed a positive correlation (Figure 2E–G). For comparative purposes, we recorded the calcium signals of anterior basolateral amygdala (aBLA)→BNST neurons (aBLA neurons retrogradely labeled from the BNST) (Figure 2H). However, the calcium activity of aBLA→BNST neurons did not show a strong correlation with the EEG signals in the intrahippocampal KA‐induced acute seizure model (Figure 2I–K). Additionally, optogenetic activation of aBLA‐BNST circuit did not generate epileptiform discharges or seizures (Figure S4, Supporting Information). Thus, these findings suggest that the activity of collateral‐projecting pBLA neurons synchronizes with epileptiform discharges (EEG signals) and increases during seizures.

### 2.4 The IC is an Important Downstream Region for pBLA Glutamatergic Neurons in Driving Seizures

**Figure 3:**
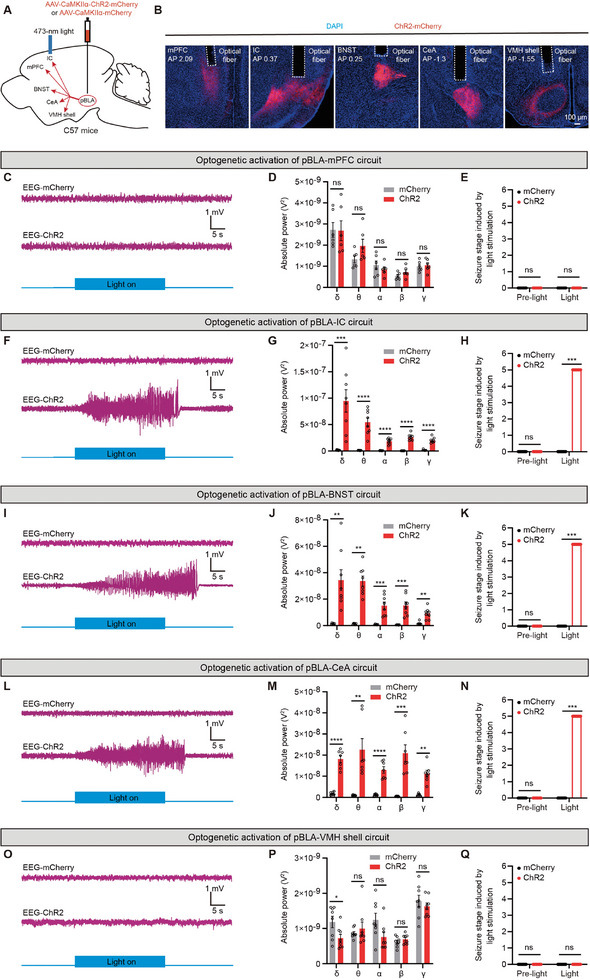
Optogenetic activation of the axon terminals of pBLA glutamatergic neurons induces seizures. A) Schematic of optogenetic activation of the pBLA glutamatergic axon terminals. B) Representative images showing the location of optical fibers in downstream brain regions of pBLA. C,F,I,L, and O) Representative EEG traces upon optogenetic activation of pBLA glutamatergic axon terminals projecting to the mPFC C), IC F), BNST I), CeA L), or VMH shell O). D,G,J,M, and P) Spectral analysis of EEG signals induced by optogenetic activation of pBLA glutamatergic axon terminals projecting to the mPFC D), IC G), BNST J), CeA M), or VMH shell P) (pBLA‐mPFC, mCherry: *n* = 6 mice, ChR2: *n* = 6 mice; pBLA‐IC, mCherry: *n* = 8 mice, ChR2: *n* = 8 mice; pBLA‐BNST, mCherry: *n* = 6 mice, ChR2: n = 8 mice; pBLA‐CeA, mCherry: *n* = 7 mice, ChR2: *n* = 7 mice; pBLA‐VMH Shell, mCherry: *n* = 8 mice, ChR2: *n* = 8 mice; two‐tailed unpaired t‐test, ns, *P* > 0.05, *P < 0.05, **P < 0.01, ***P < 0.001, ****P < 0.0001). E,H,K,N, and Q) Seizure stage induced by optogenetic activation of pBLA glutamatergic axon terminals projecting to the mPFC E), IC H), BNST K), CeA N), or VMH shell Q). (pBLA‐mPFC, mCherry: *n* = 6 mice, ChR2: *n* = 6 mice; pBLA‐IC, mCherry: *n* = 8 mice, ChR2: *n* = 8 mice; pBLA‐BNST, mCherry: *n* = 6 mice, ChR2: *n* = 8 mice; pBLA‐CeA, mCherry: *n* = 7 mice, ChR2: *n* = 7 mice; pBLA‐VMH Shell, mCherry: *n* = 8 mice, ChR2: *n* = 8 mice; Mann‐Whitney U test, ns, *P* > 0.05, ***P < 0.001). Data are presented as mean ± SEM. AAV, adeno‐associated virus; AP, anterior‐posterior axis; CaMKIIα, calcium/calmodulin‐dependent protein kinase IIα; ChR2, channelrhodopsin‐2; DAPI, 4′,6‐diamidino‐2‐phenylindole; EEG, electroencephalography; pBLA, posterior basolateral amygdala; mPFC, medial prefrontal cortex; IC, insular cortex; BNST, bed nucleus of the stria terminalis; CeA, central amygdala; VMH shell, ventromedial hypothalamus shell.

Then, we explored whether activation of pBLA glutamatergic axon terminals in downstream brain areas could induce seizures. We injected AAV‐CaMKIIα‐ChR2‐mCherry into the pBLA and bilaterally implanted optic fibers above different downstream brain regions (mPFC, IC, BNST, CeA, and VMH shell) to activate the axon terminals of pBLA glutamatergic neurons (**Figure** **3**A,B). Optogenetic activation of pBLA glutamatergic axon terminals projecting to the IC, BNST, or CeA induced palpable epileptiform discharges and seizure behaviors (Figure 3F,H,I,K,L,N), significantly increasing power across five different frequency bands of EEG waves (Figure 3G,J,M). The epileptiform discharge latency induced by activating the pBLA‐IC, pBLA‐BNST, and pBLA‐CeA circuits was 5.11 ± 0.63 s, 5.9 ± 0.78 s, and 6.14 ± 0.79 s, respectively. The discharge duration for these circuits was 39.87 ± 4.34 s (pBLA‐IC), 42.02 ± 4.44 s (pBLA‐BNST), and 37.30 ± 3.61 s (pBLA‐CeA). No significant differences were observed in either the latency or duration of epileptiform discharge among the groups (Figure S5, Supporting Information). By contrast, activation of pBLA axon terminals projecting to mPFC or VMH shell did not induce epileptiform discharges or seizure behaviors (Figure 3C,E,O,Q), nor did it significantly increase power across the five EEG frequency bands (Figure 3D,P), possibly due to fewer axonal terminals of pBLA glutamatergic neurons projecting to the mPFC or VMH shell compared to the IC, BNST, and CeA (Figure S2B,C, Supporting Information). These results indicate that optogenetic activation of pBLA glutamatergic axon terminals projecting to the IC, BNST, or CeA is sufficient to induce epileptiform discharges and seizure behaviors.

**Figure 4:**
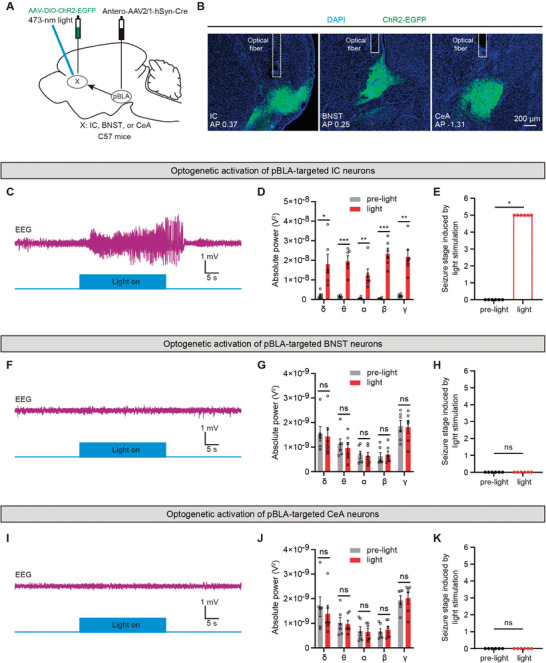
Optogenetic activation of pBLA‐targeted IC neurons induces epileptiform discharges and seizures. A) Schematic of optogenetic activation of IC, BNST, or CeA neurons that receive pBLA projections. B) Representative images showing ChR2‐EGFP expression and optic fiber placement in the IC, BNST, or CeA. C,F, and I) Representative EEG traces upon optogenetic activation of IC C), BNST F), or CeA I) neurons that receive pBLA projections. D,G, and J) Spectral analysis of EEG signals before and after light activation of IC D), BNST G), or CeA J) neurons that receive pBLA projections (*n* = 6 mice per group; two‐tailed paired t‐test, ns, *P* > 0.05, *P < 0.05, **P < 0.01, ***P < 0.001). E,H, and K) Seizure stage induced by optogenetic activation of IC E), BNST H), or CeA K) neurons that receive pBLA projections (*n* = 6 mice per group; Wilcoxon test, ns, *P* > 0.05, *P < 0.05). Data are presented as mean ± SEM. AAV, adeno‐associated virus; AP, anterior‐posterior axis; ChR2, channelrhodopsin‐2; DAPI, 4′,6‐diamidino‐2‐phenylindole; EEG, electroencephalography; EGFP, enhanced green fluorescent protein; pBLA, posterior basolateral amygdala; IC, insular cortex; BNST, bed nucleus of the stria terminalis; CeA, central amygdala.

To further determine which of the IC, BNST, or CeA is the crucial downstream region for pBLA glutamatergic neuron‐driven seizures, we selectively activated neurons in the IC, BNST, or CeA that receive projections from the pBLA. We injected anterograde trans‐synaptic Antero‐AAV2/1‐hSyn‐Cre into the pBLA, and AAV‐DIO‐ChR2‐EGFP into the IC, BNST, or CeA, followed by optic fiber implantation above the IC, BNST, or CeA (**Figure** **4**A,B). Optogenetic activation of pBLA‐targeted IC neurons resulted in significant epileptiform discharges and seizure behaviors, as well as a marked increase in the power of five different EEG frequency bands (Figure 4C–E). By contrast, optogenetic activation of downstream BNST or CeA neurons receiving pBLA projections did not induce epileptiform discharges or seizures, nor did it significantly increase the power of the five different EEG frequency bands (Figure 4F–K). These findings suggest that the IC is a key downstream region for pBLA glutamatergic neuron‐driven seizures.

**Figure 5:**
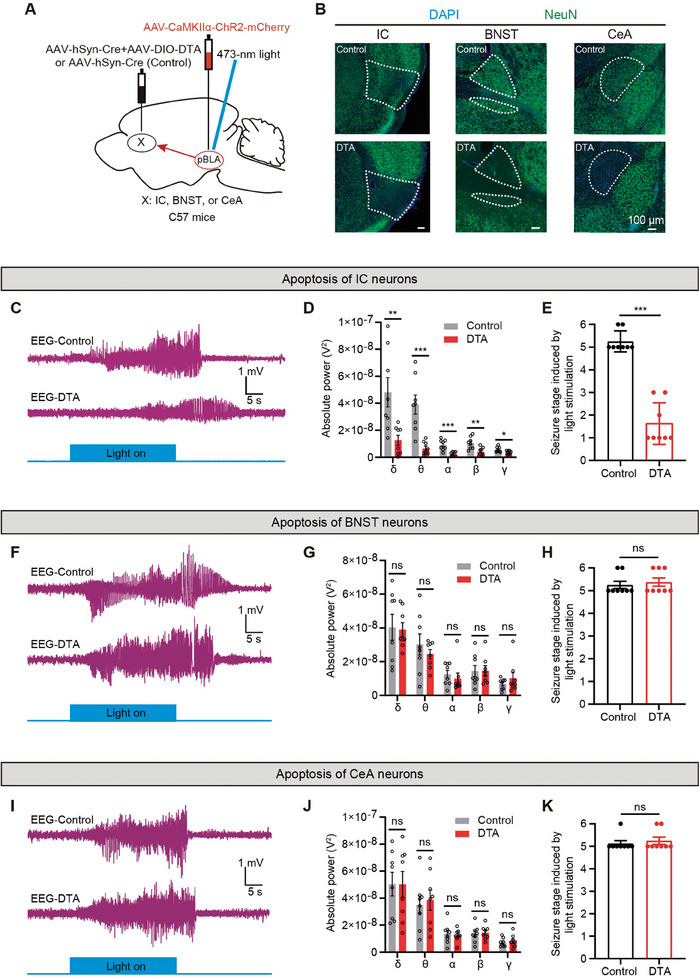
Apoptosis of IC neurons, but not BNST or CeA neurons, significantly alleviates seizures induced by the activation of pBLA glutamatergic neurons. A) Schematic of virus injection and optic fiber implantation. B) Representative images of NeuN immunofluorescence staining in the IC, BNST, or CeA regions. C,F, and I) Representative EEG traces upon optogenetic activation of pBLA glutamatergic neurons after apoptosis of IC C), BNST F), or CeA I) neurons. D,G, and J) EEG spectral analysis of the epileptiform discharges induced by optogenetic activation of pBLA glutamatergic neurons in control mice compared with mice with apoptosis of IC D), BNST G), or CeA J) neurons (*n* = 8 mice per group; two‐tailed unpaired t‐test, ns, *P* > 0.05, *P < 0.05, **P < 0.01, ***P < 0.001). E,H, and K) Seizure stage induced by optogenetic activation of pBLA glutamatergic neurons in control mice compared with mice with apoptosis of IC E), BNST H), or CeA K) neurons (*n* = 8 mice per group; Mann‐Whitney U test, ns, *P* > 0.05, ***P < 0.001). Data are presented as mean ± SEM. AAV, adeno‐associated virus; AP, anterior‐posterior axis; CaMKIIα, calcium/calmodulin‐dependent protein kinase IIα; ChR2, channelrhodopsin‐2; DAPI, 4′,6‐diamidino‐2‐phenylindole; EEG, electroencephalography; DTA, diphtheria toxin A; pBLA, posterior basolateral amygdala; IC, insular cortex; BNST, bed nucleus of the stria terminalis; CeA, central amygdala.

Next, we aimed to investigate whether silencing downstream IC neurons could alleviate seizures caused by the excitation of pBLA glutamatergic neurons. We bilaterally injected a mixture of AAV encoding diphtheria toxin A (AAV‐DIO‐DTA) and AAV‐hSyn‐Cre into the IC region of C57BL/6J mice (in the control group, only AAV‐hSyn‐Cre was bilaterally injected into the IC region), and concurrently injected AAV‐CaMKIIα‐ChR2‐mCherry into the pBLA, with optic fibers implanted above the bilateral pBLA (**Figure** **5**A). Three weeks post‐injection, mice were perfused and subjected to NeuN (a marker protein for neuronal nuclei) immunofluorescence staining. NeuN staining confirmed successful apoptosis of IC neurons (Figure 5B). The apoptosis of IC neurons mitigated the epileptiform discharges induced by the activation of pBLA glutamatergic neurons (Figure 5C), significantly reduced the power of five different EEG frequency bands during seizures (Figure 5D), and markedly mitigated the severity of seizure behaviors in the animals (Figure 5E). We also investigated the effects of inducing apoptosis of BNST or CeA neurons by injection of AAV‐DIO‐DTA and AAV‐hSyn‐Cre on seizures triggered by optogenetic activation of pBLA glutamatergic neurons (Figure 5A,B). EEG recordings and behavioral experiments showed that the apoptosis of BNST or CeA neurons did not alleviate the epileptiform discharges or reduce seizure severity induced by pBLA glutamatergic neuron activation (Figure 5F–H,I–K). These experimental results confirmed the crucial role of IC neurons in propagating pBLA‐induced epileptic activity.

### 2.5 The Loss of GABAergic Neurons in the BNST or CeA Results in Sporadic Seizures, Possibly Due to Reduced Feedback Inhibition on pBLA Glutamatergic Neurons

Surprisingly, we observed that the apoptosis of BNST or CeA neurons led to sporadic seizures in the mice, particularly when exposed to new environments (such as transferring the mice to a new cage), making the mice highly susceptible to seizures (Movies S2 and S3, Supporting Information). Given that nearly 100% of BNST and CeA neurons are inhibitory GABAergic neurons expressing *Gad2* gene (Figure S6A,B, Supporting Information), we hypothesize that the loss of GABAergic neurons in the BNST or CeA regions may reduce the inhibitory synaptic inputs into the pBLA by feedback inhibition and thereby lead to sporadic seizures. To test this hypothesis, we first injected anterograde trans‐synaptic Antero‐AAV2/1‐hSyn‐Cre into the pBLA of C57BL/6J mice and simultaneously injected AAV‐DIO‐mCherry or AAV‐DIO‐EGFP into the BNST or CeA (Figure S6C,E, Supporting Information). Interestingly, we found that neurons in the BNST or CeA regions receiving projections from the pBLA also form feedback projections to the pBLA (Figure S6D,F, Supporting Information). Since nearly all BNST or CeA neurons are inhibitory GABAergic neurons (Figure S6A,B, Supporting Information), the tracing results indicate that there are reciprocal projections between BNST^GABA+^ neurons and the pBLA neurons, as well as between CeA^GABA+^ neurons and the pBLA neurons.

**Figure 6:**
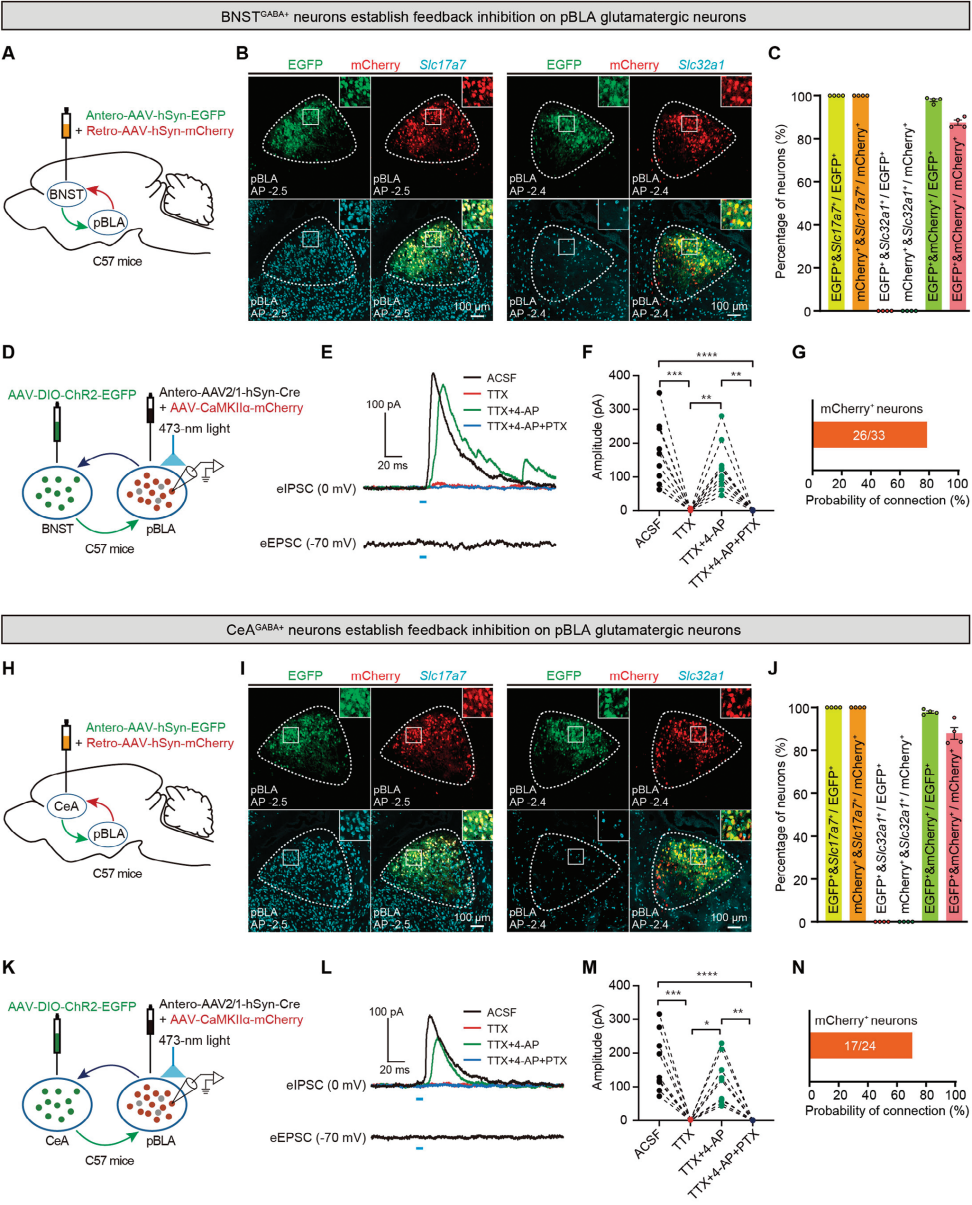
Both BNST^GABA+^ and CeA^GABA+^ neurons form feedback inhibition on pBLA glutamatergic neurons. A and H) Schematic diagrams of viral injections in the BNST or CeA regions. B and I) Representative images of cells in the pBLA region labeled with EGFP or mCherry co‐localized with *Slc17a7* and *Slc32a1*. C and J) Statistical charts of cells in the pBLA region labeled with EGFP or mCherry co‐localized with *Slc17a7* and *Slc32a1* (*Slc17a7*: *n* = 4 mice per group, 2 brain slices per mouse; *Slc32a1*: *n* = 4 mice per group, 2 brain slices per mouse). D and K) Schematic diagrams of viral injections and whole‐cell patch‐clamp electrophysiological recordings. E and L) Representative images of eIPSCs recorded from mCherry^+^ neurons in the pBLA, induced by photostimulation of ChR2‐EGFP‐expressing axon terminals from BNST or CeA neurons projecting to the pBLA. F and M) Statistical analysis of the eIPSC amplitude generated by mCherry^+^ neurons (BNST‐pBLA: *n* = 10 neurons from 5 mice; CeA‐pBLA: *n* = 9 neurons from 4 mice; Friedman test, *P < 0.05, **P < 0.01, ***P < 0.001, ****P < 0.0001). G and N) Probability of eIPSC occurrence in mCherry^+^ neurons in the pBLA (BNST‐pBLA, *n* = 33 cells from 5 mice, connection = 78.8%, 26/33; CeA‐pBLA, *n* = 24 cells from 4 mice, connection = 70.8%, 17/24). Data are presented as mean ± SEM. AAV, adeno‐associated virus; AP, anterior‐posterior axis; CaMKIIα, calcium/calmodulin‐dependent protein kinase IIα; ChR2, channelrhodopsin‐2; EGFP, enhanced green fluorescent protein; pBLA, posterior basolateral amygdala; BNST, bed nucleus of the stria terminalis; CeA, central amygdala; eIPSC, evoked inhibitory postsynaptic current; eEPSC, evoked excitatory postsynaptic current; ACSF, artificial cerebrospinal fluid; TTX, tetrodotoxin; 4‐AP, 4‐aminopyridine; PTX, picrotoxin.

To determine which type of neurons in pBLA receive feedback inhibition from the BNST^GABA+^ or CeA^GABA+^ neurons, we injected a mixture of the anterograde trans‐synaptic Antero‐AAV‐hSyn‐EGFP and the retrograde Retro‐AAV‐hSyn‐mCherry into the BNST or CeA of C57BL/6J mice (**Figure** **6**A,H). The pBLA neurons receiving projections from BNST^GABA+^ or CeA^GABA+^ neurons were labeled with EGFP, while the pBLA neurons projecting to BNST^GABA+^ or CeA^GABA+^ neurons were labeled with mCherry (Figure 6B,I). We performed FISH staining and showed that 100% of the EGFP‐ or mCherry‐labeled pBLA neurons co‐localize with *Slc17a7* (encoding VGluT1, a marker for glutamatergic neurons) but not with *Slc32a1* (encoding VGAT, a marker for GABAergic neurons) (Figure 6B,C,I,J). This indicates that BNST^GABA+^ or CeA^GABA+^ neurons form synaptic connections with glutamatergic neurons but not with GABAergic neurons in the pBLA. Additionally, ≈98% of the EGFP‐labeled pBLA glutamatergic neurons co‐expressed mCherry, and ≈87% of the mCherry‐labeled pBLA glutamatergic neurons co‐expressed EGFP (Figure 6C,J), indicating that a large proportion of the pBLA glutamatergic neurons projecting to BNST^GABA+^ or CeA^GABA+^ neurons also receive feedback projections from BNST^GABA+^ or CeA^GABA+^ neurons.

To further determine the nature of the synaptic connections between BNST^GABA+^ or CeA^GABA+^ neurons and pBLA glutamatergic neurons, we conducted whole‐cell patch‐clamp electrophysiological recording experiments. We injected a mixture of the anterograde trans‐synaptic Antero‐AAV2/1‐hSyn‐Cre and AAV‐CaMKIIα‐mCherry into the pBLA, and the AAV‐DIO‐ChR2‐EGFP into the BNST or CeA in the same mice. This strategy enables us to simultaneously label pBLA glutamatergic neurons with mCherry and the BNST or CeA neurons that received projections from the pBLA with ChR2‐EGFP (Figure 6D,K). Three weeks after viral expression, we performed patch‐clamp electrophysiological recording on acute brain slices and found light‐stimulation of the axon terminals from BNST or CeA to the pBLA evoked inhibitory postsynaptic currents (eIPSCs), but not excitatory postsynaptic currents (eEPSCs), in over 70% of the mCherry‐labeled glutamatergic neurons in the pBLA (Figure 6E,G,L,N). Additionally, the pBLA glutamatergic neurons exhibited monosynaptic inhibitory responses to optogenetic activation of the axon terminals of BNST^GABA+^ or CeA^GABA+^ neurons, as evidenced by the following: the recorded eIPSCs were blocked by the voltage‐gated sodium channel blocker tetrodotoxin (TTX, 1 µM), subsequently restored by the potassium channel blocker 4‐aminopyridine (4‐AP, 100 µM), and abolished by the GABA_A_ receptor antagonist picrotoxin (PTX, 100 µM) (Figure 6E,F,L,M). These results indicate that the GABAergic neurons in the BNST or CeA, which receive projections from the pBLA, form feedback inhibitory monosynaptic inputs to pBLA glutamatergic neurons (Figure 6B,C,I,J). Combining the results of viral tracing, FISH, and patch‐clamp electrophysiological recording, we demonstrated that BNST^GABA+^ or CeA^GABA+^ neurons form feedback inhibition on pBLA glutamatergic neurons.

**Figure 7:**
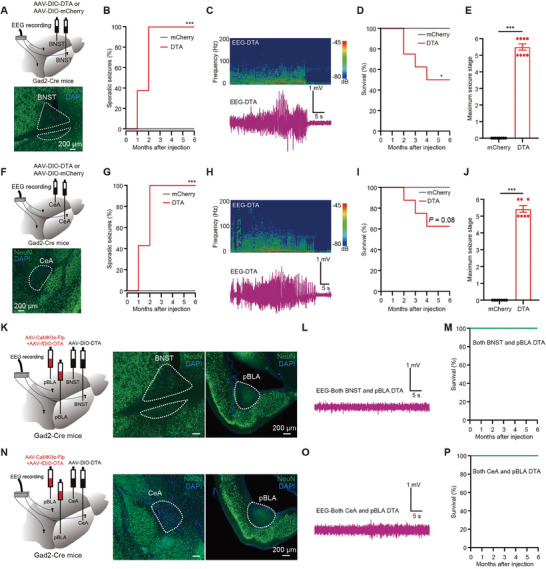
Deletion of GABAergic neurons in BNST or CeA results in sporadic seizures, possibly due to a reduction in feedback inhibition on pBLA glutamatergic neurons. A and F) Upper: Schematic of bilateral ablation of BNST or CeA GABAergic neurons and EEG recordings. Lower: Representative images of NeuN staining for BNST or CeA. B and G) Percentage of mice with ablation of BNST or CeA GABAergic neurons displaying sporadic seizures (BNST‐mCherry, *n* = 8 mice; BNST‐DTA, *n* = 8 mice; CeA‐mCherry, *n* = 7 mice; CeA‐DTA, *n* = 7 mice; Log‐rank test, ***P < 0.001). C and H) Representative EEG power spectrum and EEG traces recorded in mice with ablation of BNST or CeA GABAergic neurons. D and I) Survival rate of mice shown in A and F) (BNST‐mCherry, *n* = 8 mice; BNST‐DTA, *n* = 8 mice; CeA‐mCherry, *n* = 7 mice; CeA‐DTA, *n* = 7 mice; Log‐rank test, *P < 0.05). E and J) Maximum seizure stage recorded in mice with ablation of BNST or CeA GABAergic neurons (BNST‐mCherry, *n* = 8 mice; BNST‐DTA, *n* = 8 mice; CeA‐mCherry, *n* = 7 mice; CeA‐DTA, *n* = 7 mice; Mann‐Whitney U test, ***P < 0.001). K and N) Left: Schematic of bilateral ablation of BNST or CeA GABAergic neurons together with pBLA glutamatergic neurons and EEG recordings. Right: Representative images of NeuN staining for BNST, CeA, and pBLA. L and O) Representative EEG traces recorded in mice with ablation of BNST or CeA GABAergic neurons together with pBLA glutamatergic neurons. M and P) Survival rate of mice shown in K and N) (*n* = 8 mice respectively). Data are presented as mean ± SEM. AAV, adeno‐associated virus; CaMKIIα, calcium/calmodulin‐dependent protein kinase IIα; DAPI, 4′,6‐diamidino‐2‐phenylindole; EEG, electroencephalography; DTA, diphtheria toxin A; pBLA, posterior basolateral amygdala; BNST, bed nucleus of the stria terminalis; CeA, central amygdala.

To further investigate the role of feedback inhibition from BNST^GABA+^ or CeA^GABA+^ neurons to pBLA glutamatergic neurons in seizures, we employed a loss‐of‐function strategy by injecting AAV‐DIO‐DTA into the BNST or CeA of Gad2‐Cre mice to induce apoptosis of GABAergic neurons (**Figure** **7**A,F). One month after DTA injection, ≈40% of the mice exhibited sporadic, recurrent epileptiform discharges and seizures; two months after DTA injection, all mice showed sporadic epileptiform discharges and seizures (Figure 7B,C,G,H). Four months after DTA injection, the mortality rate due to seizures was ≈40% (Figure 7D,E,I,J). Importantly, in *Gad2*‐Cre mice where both BNST^GABA+^ neurons and pBLA glutamatergic neurons, or CeA^GABA+^ neurons and pBLA glutamatergic neurons, were simultaneously ablated (Figure 7K,N), no epileptiform discharges, seizures, or deaths were observed (Figure 7L,M,O,P). These findings suggest that the loss of BNST^GABA+^ or CeA^GABA+^ neurons leads to sporadic seizures, possibly due to reduced feedback inhibition on pBLA glutamatergic neurons following the apoptosis of GABAergic neurons in the BNST or CeA, resulting in abnormal excitation of pBLA glutamatergic neurons and subsequent epileptiform discharges and seizures. In conclusion, these results indicate that GABAergic neurons in the BNST and CeA may act as “brakes” by providing feedback inhibition to prevent excessive activity of pBLA glutamatergic neurons, thereby inhibiting epileptiform discharges and seizures under physiological conditions.

### 2.6 Seizures Induced by Activation of pBLA Axon Terminals in the BNST or CeA may be Mediated Through Antidromic Activation of pBLA Glutamatergic Neurons

It is intriguing that optogenetic activation of pBLA glutamatergic axon terminals projecting to the BNST or CeA also induced seizures. Given the significant collateralization of pBLA glutamatergic projections to BNST, CeA, and IC (Figure 2A–C; Figure S3A–I, Supporting Information), it is possible that optogenetic stimulation of axon terminals in the BNST or CeA antidromically activates pBLA somas, resulting in seizures. Given the importance of this issue in interpreting our ChR2 terminal stimulation studies, we examined whether optogenetic stimulation of pBLA axon terminals could antidromically activate pBLA neuronal somas. We injected a mixture of AAV‐CaMKIIα‐Cre, AAV‐DIO‐GCaMP6s, and AAV‐DIO‐ChR2‐mCherry (or AAV‐DIO‐mCherry) into the pBLA. An optic fiber was implanted above the BNST to activate the pBLA neuronal axon terminals, and another optic fiber was implanted in the pBLA to monitor the activity of pBLA neuronal somas in vivo (Figure S7A, Supporting Information). We simultaneously monitored the activity of pBLA neuronal somas using in vivo fiber photometry during optogenetic stimulation of pBLA axon terminals. Using the onset of light stimulation as the starting point (0 seconds), we plotted the change in calcium signals of pBLA neuronal somas and calculated the area under the curve (AUC, with areas above 0 considered as positive and areas below 0 considered as negative) during light stimulation. Our results showed a sharp increase in pBLA neuronal soma calcium activity upon optogenetic stimulation of pBLA axon terminals (Figure S7B,C, Supporting Information). These data suggest that optogenetic stimulation of pBLA neuronal axon terminals in the BNST may antidromically activate pBLA neuronal somas, thereby driving seizures.

### 2.7 Activation of Excitatory Inputs from vCA1 to pBLA Neurons Results in Seizures

**Figure 8:**
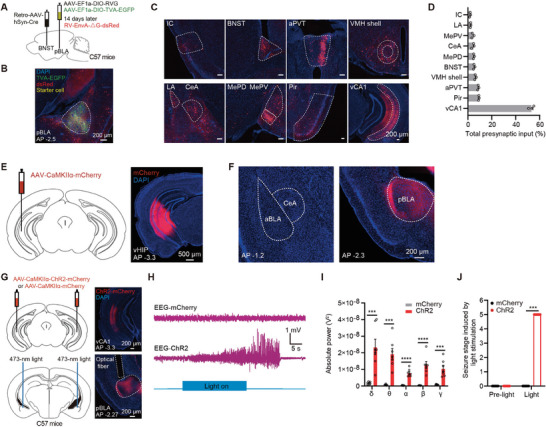
Regulation of pBLA glutamatergic neurons by vCA1 contributes to seizures. A) Schematic of monosynaptic retrograde tracing from pBLA neurons projecting to the BNST (pBLA→BNST). B) Representative image showing starter cells labeled in pBLA. C) Representative images of brain regions projecting to pBLA→BNST neurons. D) Quantification of presynaptic inputs in C) (*n* = 3 mice). E) Schematic of viral injection and expression in the vHIP (*n* = 3 mice). F) Distribution of mCherry‐labeled excitatory axon terminals from the vHIP in the amygdalar region. G) Left: Schematic of optogenetic activation of vCA1‐pBLA circuit. Right: ChR2‐mCherry expression in vCA1 and the location of optical fiber in pBLA. H) Representative EEG traces induced by optogenetic activation of vCA1‐pBLA circuit. I) EEG spectral analysis of the epileptiform discharges induced by optogenetic activation of vCA1‐pBLA circuit (*n* = 7 mice per group; two‐tailed unpaired t‐test, ***P < 0.001, ****P < 0.0001). J) Seizure stage induced by optogenetic activation of vCA1‐pBLA circuit (*n* = 7 mice per group; Mann‐Whitney U test, ***P < 0.001). Data are presented as mean ± SEM. AAV, adeno‐associated virus; AP, anterior‐posterior axis; CaMKIIα, calcium/calmodulin‐dependent protein kinase IIα; ChR2, channelrhodopsin‐2; DAPI, 4′,6‐diamidino‐2‐phenylindole; EEG, electroencephalography; EGFP, enhanced green fluorescent protein; RV, rabies virus; EnvA, envelope protein of the avian sarcoma‐leukosis virus, subtype A; RVG, rabies virus glycoprotein; TVA, retroviral avian rabies receptor; ΔG, deficient glycoprotein; pBLA, posterior basolateral amygdala; aBLA, anterior basolateral amygdala; IC, insular cortex; BNST, bed nucleus of the stria terminalis; CeA, central amygdala; aPVT, anterior paraventricular thalamus; LA, lateral amygdala; MePD, medial amygdaloid nucleus, posterodorsal part; MePV, medial amygdaloid nucleus, posteroventral part; Pir, piriform cortex; VMH shell, ventromedial hypothalamus shell; vCA1, ventral CA1; vHIP, ventral hippocampus.

Furthermore, to identify the upstream inputs to pBLA glutamatergic neurons responsible for seizures, we employed a circuit‐specific monosynaptic retrograde tracing strategy using rabies virus (RV). We injected Retro‐AAV‐hSyn‐Cre into the BNST, one of the primary downstream regions of pBLA, and injected Cre‐dependent RV helper viruses (AAV‐EF1α‐DIO‐RVG and AAV‐EF1α‐DIO‐TVA‐EGFP, mixed 1:1; RVG, rabies virus glycoprotein; TVA, retroviral avian rabies receptor) into the pBLA. Two weeks later, we injected the G‐protein‐deficient rabies virus (RV‐EnvA‐ΔG‐dsRed; EnvA, envelope protein of the avian sarcoma‐leukosis virus, subtype A; ΔG, deficient glycoprotein) into the pBLA (**Figure** **8**A,B) and examined the expression of RV‐EnvA‐ΔG‐dsRed one week after injection. We found that pBLA glutamatergic neurons receive substantial inputs from the ventral hippocampal CA1 (vCA1) region (Figure 8C,D). Additionally, we injected AAV‐CaMKIIα‐mCherry into the ventral hippocampus (vHIP) for anterograde tracing (Figure 8E) and found extensive excitatory axon terminal projections from the vHIP to the pBLA, but not to other amygdalar nuclei such as the aBLA and CeA (Figure 8F). These retrograde and anterograde tracing results indicate that the pBLA receives substantial excitatory inputs from the vCA1.

To test whether activating the vCA1‐pBLA neural circuit is sufficient to induce seizures, we injected AAV‐CaMKIIα‐ChR2‐mCherry or AAV‐CaMKIIα‐mCherry into the vCA1 of C57BL/6J mice and implanted optical fibers above the pBLA (Figure 8G). Optogenetic activation of the vCA1‐pBLA neural circuit induced epileptiform discharges and seizure behaviors (Figure 8H–J). By contrast, although pBLA neurons also receive synaptic inputs from the piriform cortex (Pir) and anterior paraventricular thalamus (aPVT) (Figure 8D), optogenetic activation of the excitatory Pir‐pBLA or aPVT‐pBLA neural circuits did not induce epileptiform discharges or seizure behaviors (Figure S8, Supporting Information). These results demonstrate that excitatory inputs from vCA1 to the pBLA play a critical role in the onset of seizures.

### 2.8 Ablation of pBLA Glutamatergic Neurons Significantly Alleviates both Acute and Chronic Seizures in a Mouse Model of Temporal Lobe Epilepsy

All the above results demonstrated the important role of pBLA glutamatergic neurons in driving epileptic seizures. Finally, we wanted to explore the role of pBLA glutamatergic neurons in TLE. We investigated whether silencing glutamatergic neurons in the pBLA could alleviate epileptic seizures in a mouse model of TLE induced by intrahippocampal KA injection. The intrahippocampal administration of KA induced acute status epilepticus (SE), followed by the typical emergence of spontaneous recurrent seizures after several weeks. Using this model, we explored the role of pBLA in controlling TLE by selectively targeting and ablating pBLA glutamatergic neurons.

**Figure 9:**
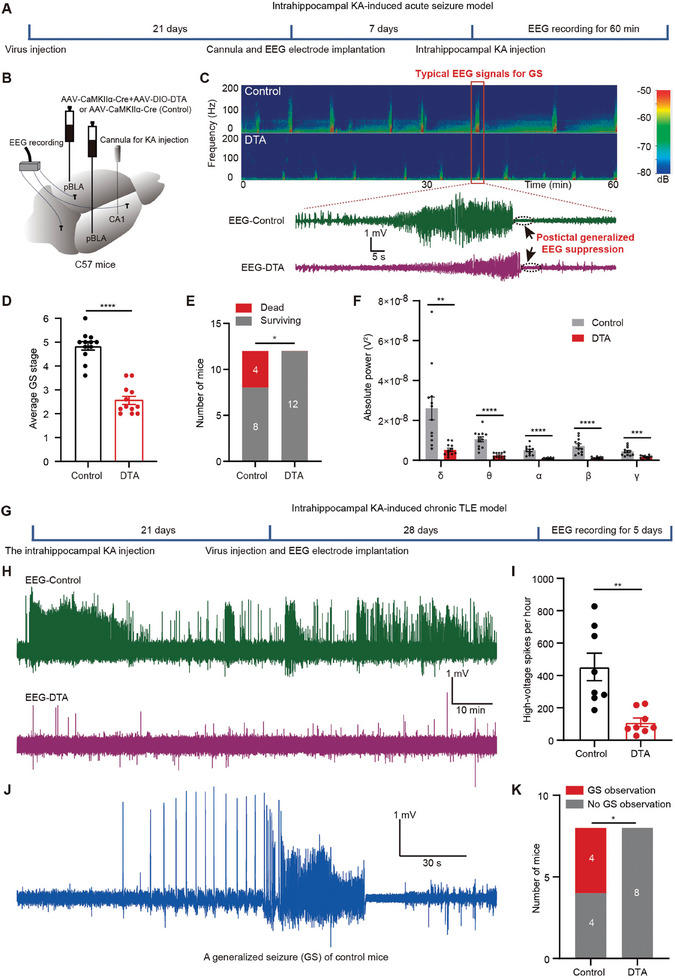
Ablation of pBLA glutamatergic neurons significantly alleviates acute and chronic seizures in intrahippocampal KA‐induced TLE model. A) Schematic of the intrahippocampal KA‐induced acute seizure model. B) Schematic of EEG recordings. C) Upper: Representative EEG power spectrum in the acute seizure model. Lower: Enlarged view of typical EEG traces for GS from the red box in the upper panel. D) Average GS stage within 60 min in the acute seizure model (*n* = 12 mice per group; Mann‐Whitney U test, ****P < 0.0001). E) Number of dead and surviving mice in the acute seizure model (*n* = 12 mice per group; Chi‐square test, *P < 0.05). F) Spectral analysis of EEG signals in the acute seizure model (*n* = 12 mice per group; two‐tailed unpaired *t*‐test, **P < 0.01, ***P < 0.001, ****P < 0.0001). G) Schematic of the intrahippocampal KA‐induced chronic TLE model. H) Typical spontaneous high‐voltage spikes recorded in the chronic TLE model. I) Quantification of spontaneous high‐voltage spikes (> 0.5 mV) in the chronic TLE model (*n* = 8 mice per group; two‐tailed unpaired t test, **P < 0.01). J) Typical EEG signals for a spontaneous generalized seizure (GS) of control mice in the chronic TLE model. K) Number of mice that developed GSs in the chronic TLE model (*n* = 8 mice per group; Chi‐square test, *P < 0.05). Data are presented as mean ± SEM. AAV, adeno‐associated virus; CaMKIIα, calcium/calmodulin‐dependent protein kinase IIα; DTA, diphtheria toxin A; EEG, electroencephalography; KA, kainic acid; pBLA, posterior basolateral amygdala; GS, generalized seizure; TLE, temporal lobe epilepsy.

To assess whether ablation of pBLA glutamatergic neurons could mitigate KA‐induced acute seizures (**Figure** **9**A), we bilaterally injected a mixture of AAV‐DIO‐DTA and AAV‐CaMKIIα‐Cre into the pBLA (Figure 9B). Four weeks after virus injection, we observed a loss of NeuN‐positive cells in the pBLA in mice bilaterally injected with DTA, but not in control mice injected with AAV‐CaMKIIα‐Cre only (Figure S9, Supporting Information). Subsequent to KA administration into the hippocampal CA1 region via an implanted cannula guide, mice were monitored for 60 min to evaluate both seizure behaviors and EEG signals from the skull surface (Figure 9A). In this study, generalized seizures (GSs) were identified by characteristic EEG patterns. GS discharges were characterized by brief high‐amplitude EEG signals followed by flattened EEG signals (postictal generalized EEG suppression, PGES) (Figure 9C), a pattern typically occurring during severe seizures. All GS events in each mouse over a 60 min period were rated according to the modified Racine scale, and the average of these ratings was taken as the GS grade for that mouse. Mice subjected to pBLA ablation displayed significant reductions in both the EEG power spectrum and severity of GS (Figure 9C,D). pBLA ablation also markedly lowered fatal outcomes during acute seizure episodes (Figure 9E). Spectral analysis of EEG signals indicated a substantial reduction in power across the five types of brain waves (Figure 9F). Collectively, these findings demonstrate that the ablation of pBLA glutamatergic neurons is a potent intervention for suppressing KA‐induced acute seizures and reducing the risk of severe seizure‐related death.

Next, we explored whether ablation of pBLA glutamatergic neurons could suppress KA‐induced chronic recurrent spontaneous seizures. In brief, we injected KA into the hippocampus, bilaterally introduced AAV‐DIO‐DTA and AAV‐CaMKIIα‐Cre for pBLA ablation, and placed electrodes on the skull surface for EEG recordings. Seven weeks after KA injection, spontaneous high‐voltage spikes (> 0.5 mV) were evaluated in mice during over an 8 h observation period each day for 5 days (Figure 9G). Ablation of the pBLA glutamatergic neurons significantly reduced the number of spontaneous high‐voltage spikes (Figure 9H,I). GSs were also absent in mice with pBLA ablation (Figure 9J,K). These findings demonstrate that pBLA ablation effectively mitigates seizures in chronic TLE models.

## 3 Discussion

Seizures manifest as sudden and transient symptoms arising from excessive and synchronized neuronal activity in the brain.^[^ ^25^ ^]^ Activation of pBLA glutamatergic neurons, which receive excitatory inputs from the vCA1 (Figure 8C–F), resulted in pronounced epileptiform discharges and grade 5–6 seizures, along with other seizure symptoms, including pupillary dilation and foaming at the mouth (Figure 1). These symptoms resemble the tonic‐clonic seizures clinically observed in epilepsy patients.^[^ ^26^, ^27^, ^28^ ^]^ pBLA glutamatergic neurons collaterally project to multiple brain regions, including the IC, BNST, and CeA (Figure 2C). Activation of pBLA‐targeted IC neurons, but not pBLA‐targeted BNST or CeA neurons, induced seizures (Figure 4). However, optogenetic activation of pBLA glutamatergic axon terminals in the IC, BNST, or CeA all induced seizure activities (Figure 3F–N). It has been reported that optogenetic stimulation of axon terminals can generate antidromic conduction.^[^ ^29^ ^]^ Indeed, we found that optogenetic activation of pBLA glutamatergic axon terminals in the BNST antidromically activates pBLA neuronal somas (Figure S7, Supporting Information), thereby inducing seizures.

Insular epilepsy shares many clinical and EEG features with TLE.^[^ ^30^ ^]^ The involvement of IC in TLE has gradually been recognized with the widespread use of stereoelectroencephalography (SEEG), and the ictal temporal discharges often invade the insula.^[^ ^31^ ^]^ We found that pBLA glutamatergic neurons located in the temporal lobe directly project to IC neurons (Figure S2, Supporting Information), and the apoptosis of IC neurons mitigated the epileptiform discharges and seizure stages induced by the activation of pBLA glutamatergic neurons (Figure 5C–E). This suggests that the IC is an effective downstream target of pBLA glutamatergic neurons for seizure propagation. Future investigations are warranted to elucidate the role of IC neurons in acute or chronic TLE models.

Previous studies have reported that the BNST and CeA are predominantly composed of inhibitory GABAergic neurons.^[^ ^32^, ^33^, ^34^ ^]^ The BNST and CeA serve as the principal downstream targets of the pBLA, and we confirmed that nearly 100% of BNST and CeA neurons are inhibitory GABAergic neurons that express *Gad2* gene (Figures S2 and S6A,B, Supporting Information). The pBLA belongs to the nuclei of the subcortical group of the amygdaloid complex, and consists of mostly glutamatergic projection neurons and a few GABAergic interneurons.^[^ ^23^ ^]^ Our results revealed that BNST^GABA+^ and CeA^GABA+^ neurons form feedback inhibition on pBLA glutamatergic neurons by integrating multiple approaches including circuit tracing, FISH and in vitro electrophysiology (Figure 6; Figure S6, Supporting Information). Loss of GABAergic neurons in the BNST or CeA resulted in sporadic seizures (Figure 7A–J). Moreover, concurrent ablation of BNST^GABA+^ or CeA^GABA+^ neurons with pBLA glutamatergic neurons blocked the occurrence of epileptiform discharges and seizures in mice (Figure 7K–P), suggesting that the loss of GABAergic neurons in the BNST or CeA results in sporadic seizures, possibly due to a reduction in feedback inhibition on pBLA glutamatergic neurons. This is consistent with findings that reduced feedback inhibition promotes epileptic seizures in cortical circuits.^[^ ^35^, ^36^, ^37^ ^]^ However, feedback inhibition from reticular thalamic GABAergic neurons in the cortico‐thalamic circuit can promote seizure responses.^[^ ^38^ ^]^ Future studies are needed to determine the precise circuitry mechanisms of BNST^GABA+^ and CeA^GABA+^ neurons in regulating epileptic seizures in TLE.

The hippocampus has been reported to be closely associated with seizures in TLE.^[^ ^39^, ^40^, ^41^, ^42^, ^43^, ^44^ ^]^ Virus tracing results show that the pBLA receives substantial excitatory inputs from vCA1, and optogenetic activation of the vCA1‐pBLA neural circuit can lead to seizures (Figure 8). Although pBLA receives synaptic inputs from the Pir and aPVT, which are primarily composed of glutamatergic neurons,^[^ ^45^, ^46^ ^]^ optogenetic activation of the excitatory Pir‐pBLA or aPVT‐pBLA circuits did not result in seizures (Figure S8, Supporting Information). This may be attributed to the relatively weaker excitatory inputs from the Pir or aPVT to the pBLA, compared to those from vCA1 (Figure 8C,D), which are insufficient to trigger seizures.

Epileptogenesis, the development of epilepsy, can result from malfunction of neural circuits in brain regions of initial insult or choke points that play a key role in abnormal circuits and are remote from the initial dysfunction.^[^ ^35^ ^]^ The DTA‐mediated ablation to eliminate pBLA glutamatergic neurons significantly alleviates seizures in both intrahippocampal KA‐induced acute and chronic seizure models (Figure 9). These results suggest that in the amygdaloid complex, the pBLA functions as a critical choke point in the TLE model, with pBLA glutamatergic neurons located remote from the hippocampus, the site of initial insult in KA‐induced TLE. To more accurately emulate the physiological conditions, we also used chemogenetics to examine the role of the pBLA in seizure control in the intrahippocampal KA‐induced acute seizure model (Figure S10A,B, Supporting Information). Chemogenetic inhibition of pBLA glutamatergic neurons resulted in significant reductions in the EEG power spectrum and the severity of generalized seizures (GSs) (Figure S10C–F, Supporting Information). However, the decrease in α and γ EEG power following clozapine N‐oxide (CNO) injection was significantly less pronounced compared to the reduction of EEG power after DTA‐mediated ablation of these neurons (Figure S10F, Supporting Information). This is possibly because chemogenetic inhibition may not fully suppress the epileptiform discharges of pBLA glutamatergic neurons induced by intrahippocampal KA injection. Therefore, we mainly utilized DTA‐mediated ablation in this study to selectively eliminate specific neuronal populations to assess their impact on seizure activity.

Here, we highlight the pBLA as a pivotal nucleus within the amygdaloid complex for regulating epileptic seizures in TLE. However, several intriguing questions remain unanswered. The apoptosis of pBLA neurons significantly alleviates but does not completely block epileptiform discharges, suggesting that other nuclei may also mediate the propagation of epileptiform discharges, warranting further research. While our use of one rodent model of TLE provides valuable insights into the circuit mechanisms of pBLA neurons in seizure genesis and propagation, it remains to be determined whether the pBLA circuits are critical for acute or chronic epileptic seizures in other TLE models. Previous studies have suggested that sex differences exist in epilepsy,^[^ ^47^, ^48^, ^49^ ^]^ with male and female rodents exhibiting varying susceptibilities to different modeling methods.^[^ ^50^, ^51^ ^]^ In our study, we focused on male mice for the experiments; therefore, the role of pBLA glutamatergic neurons in seizure genesis or seizure propagation in female mice requires further investigation.

In this study, our findings revealed previously unrecognized pBLA circuits and the collateral neural network involved in epileptic seizures. We also provided experimental evidence of the pivotal role of the pBLA, a downstream region of the ventral hippocampal CA1, as a crucial choke point in seizure generation in TLE (Figure S11, Supporting Information). These results have the potential to catalyze the development of more precise and effective interventions targeting neural circuits for the treatment of TLE.

## 4 Conclusion 

In summary, we highlight the pBLA as a pivotal nucleus in the amygdaloid complex for regulating epileptic seizures in TLE. Optogenetic activation of pBLA glutamatergic neurons, which collaterally project to multiple brain regions including the IC, BNST, and CeA, induces severe epileptic seizures and even death of mice. The IC is a crucial downstream region for the seizure propagation induced by pBLA glutamatergic neuron activation, while long‐projecting GABAergic neurons in the BNST and CeA provide feedback inhibition to balance the activity of pBLA glutamatergic neurons. Ablation of pBLA glutamatergic neurons significantly alleviates both acute and chronic seizures in intrahippocampal KA‐induced mouse model of TLE. These findings offer novel circuit mechanisms of pBLA in regulating epileptic seizures in TLE and have the potential to catalyze the development of more precise and effective interventions targeting neural circuits for the treatment of TLE.

## 5 Experimental Section

### Animals

All animal care and experimental procedures were conducted under the guidelines of the Animal Care and Use Committee of the Animal Facility at Zhejiang University and approved by the Laboratory Animal Welfare and Ethics Committee of Zhejiang University (authorization: ZJU20220384). There was gender differences in different types of epilepsy,^[^ ^47^, ^48^, ^49^ ^]^ with male and female rodents exhibiting varying susceptibilities to different epilepsy modeling methods.^[^ ^50^, ^51^ ^]^ Furthermore, female animals experience hormonal fluctuations throughout their estrous cycle, which can influence both behavior and physiological responses. This variability may confound experimental results, complicating the ability to draw clear conclusions. To minimize the influence of sex differences and hormonal fluctuations on this experimental results, experiments were performed on adult male C57BL/6J, Gad2‐Cre (Jackson Laboratory, Strain #01 9022), and vGluT2‐Cre (Jackson Laboratory, Strain#01 6963) mice (8—14 weeks). Mice were randomly assigned to either the control group or the experimental group. They were housed in a controlled environment with a constant temperature of 22 ± 1 °C and maintained under a 12 h light/12 h dark cycle (lights on from 7 am to 7 pm). Standard chow and water were provided ad libitum, and relative humidity was kept between 30% and 50%. The mice were placed in individually ventilated cages (VM370B, Suhangtech, China) with dimensions of 370 × 157 × 180 mm, accommodating no more than six mice per cage. Bedding was changed weekly, and no additional environmental enrichment was provided. All behavioral experiments were conducted during the light cycle. Prior to surgery, the mice were group‐housed; however, after the surgical procedures, all experimental mice with implants for EEG recordings were housed individually. The total number of mice used in the study was 396.

### Viruses

AAV2/9‐CaMKIIα‐ChR2‐mCherry, AAV2/9‐CaMKIIα‐hM4Di‐mCherry, AAV2/9‐CaMKIIα‐mCherry, AAV2/9‐EF1α‐DIO‐EGFP, AAV2/9‐EF1α‐DIO‐mCherry, AAV2/9‐EF1α‐DIO‐GCaMP6s, AAV2/9‐hSyn‐Cre, AAV2/9‐EF1α‐DIO‐hChR2(H134R)‐EGFP, Retro‐AAV2/2‐hSyn‐Cre, Retro‐AAV2/2‐hSyn‐EGFP, and Retro‐AAV2/2‐hSyn‐mCherry were purchased from OBiO Technology Co., Ltd (Shanghai, China).

AAV2/9‐CaMKIIα‐Cre, AAV2/9‐EF1α‐DIO‐DTA, AAV2/9‐CaMKIIα‐Flp, and AAV2/9‐EF1α‐fDIO‐DTA were purchased from BrainVTA Technology Co., Ltd (Wuhan, China).

AAV2/9‐EF1α‐DIO‐TVA‐EGFP, AAV2/9‐EF1α‐DIO‐RVG, and RV‐EnvA‐ΔG‐dsRed were purchased from Brain Case Co., Ltd (Shenzhen, China).

Antero‐AAV2/1‐hSyn‐Cre and Antero‐AAV2/1‐hSyn‐EGFP were purchased from Taitool Bioscience Co., Ltd (Shanghai, China).

All virus titers were >10^12^ viral genomes /mL.

### Stereotactic Surgery

Mice were anesthetized with pentobarbital sodium (50 mg kg^−1^, intraperitoneal injection (i.p.)) and placed on a stereotaxic apparatus (RWD Life Technology, China). Their eyes were covered with ophthalmic ointment to maintain moisture. Body temperature was maintained at 35—37 °C using a heating pad. After shaving their hair and cleaning the incision site with medical alcohol, the scalps of the mice were incised to expose the skull. Small craniotomy holes (≈1 mm diameter) were drilled in the skull above the target areas to facilitate virus injection. The virus was injected with a 10‐µl microliter syringe (Gaoge Industry and Trade Co., Ltd., China) connected to a glass micropipette with a 10–15‐mm diameter tip. The speed (40 nl min^−1^) and volume of the virus injection were controlled using a syringe pump (KD Scientific, 78–8130, USA).

For optogenetic activation of pBLA or aBLA glutamatergic neurons, AAV‐CaMKIIα‐ChR2‐mCherry (80 nl per side) was injected into the bilateral pBLA (AP = −2.40 mm; ML = ±3.30 mm; DV = −4.95 mm) or aBLA (AP = −1.0 mm; ML = ±3.30 mm; DV = −4.85 mm), and optical fibers were bilaterally implanted above the pBLA (AP = −2.40 mm; ML = ±3.30 mm; DV = −4.65 mm) or aBLA (AP = −1.0 mm; ML = ±3.30 mm; DV = −4.55 mm).

To assess the collateral projections emanating from pBLA neurons, a 3:1 volume mixture of Retro‐AAV‐hSyn‐Cre and AAV‐DIO‐EGFP (80 nl) was injected into the unilateral BNST (AP = +0.25 mm; ML = 1.00 mm; DV = −4.05 mm), and AAV‐DIO‐mCherry (80 nl) was injected into the unilateral pBLA (AP = −2.40 mm; ML = 3.30 mm; DV = −4.95 mm).

For dual retrograde injection into the BNST, CeA, and IC, Retro‐AAV‐hSyn‐mCherry or EGFP (80 nl per side) was injected into the BNST (AP = +0.25 mm; ML = ±1.0 mm; DV = −4.05 mm), Retro‐AAV‐hSyn‐mCherry or EGFP (80 nl per side) was injected into the CeA (AP = −1.30 mm; ML = ±2.90 mm; DV = −4.50 mm), and Retro‐AAV‐hSyn‐EGFP (80 nl per side) was injected into the IC (AP = +0.40 mm; ML = ±3.80 mm; DV = −4.00 mm).

For recording calcium signals of the pBLA or aBLA in a projection‐specific manner, Retro‐AAV‐hSyn‐Cre (60 nl) was injected into the BNST (AP = +0.25 mm; ML = ±1.0 mm; DV = −4.05 mm), and AAV‐DIO‐GCaMP6s (70 nl) was injected into the pBLA (AP = −2.40 mm; ML = ±3.30 mm; DV = −4.95 mm) or aBLA (AP = −0.95 mm; ML = ±3.30 mm; DV = −4.90 mm). Optical fibers were implanted in the pBLA (AP = −2.40 mm; ML = ±3.30 mm; DV = −4.75 mm) or aBLA (AP = −0.95 mm; ML = ±3.30 mm; DV = −4.70 mm).

For optogenetic activation of the aBLA‐BNST circuit, AAV‐CaMKIIα‐ChR2‐mCherry (80 nl per side) was injected into the bilateral aBLA (AP = −0.95 mm; ML = ±3.30 mm; DV = −4.90 mm), and optical fibers were bilaterally implanted above the BNST (AP = +0.25 mm; ML = ±1.67 mm; DV = −3.40 mm, with a 10° angle toward midline).

For optogenetic activation of the axon terminals of pBLA glutamatergic neurons, AAV‐CaMKIIα‐ChR2‐mCherry (80 nl per side) was bilaterally injected into the bilateral pBLA (AP = −2.40 mm; ML = ±3.30 mm; DV = −4.95 mm), and optical fibers were bilaterally implanted above the BNST (AP = +0.25 mm; ML = ±1.67 mm; DV = −3.40 mm, with a 10° angle toward midline), CeA (AP = −1.30 mm; ML = ±2.90 mm; DV = −4.20 mm), IC (AP = +0.40 mm; ML = ±3.80 mm; DV = −3.40 mm), VMH shell (AP = −1.60 mm; ML = ±1.45 mm; DV = −4.90 mm, with a 10° angle toward midline), or mPFC (AP = +2.0 mm; ML = ±0.75 mm; DV = −1.90 mm, with a 10° angle toward midline).

For selectively activating neurons in the IC, BNST, or CeA that receive projections from the pBLA, anterograde trans‐synaptic Antero‐AAV2/1‐hSyn‐Cre (100 nl per side) was bilaterally injected into the pBLA (AP = −2.40 mm; ML = ±3.30 mm; DV = −4.95 mm), and AAV‐DIO‐ChR2‐EGFP (100 nl per side) into the IC (AP = +0.40 mm; ML = ±3.80 mm; DV = −3.80 mm), BNST (AP = +0.25 mm; ML = ±1.0 mm; DV = −4.05 mm), or CeA (AP = −1.30 mm; ML = ±2.90 mm; DV = −4.50 mm), followed by optic fiber implantation above the bilateral IC (AP = +0.40 mm; ML = ±3.80 mm; DV = −3.40 mm), BNST (AP = +0.25 mm; ML = ±1.67 mm; DV = −3.40 mm, with a 10° angle toward midline), or CeA (AP = −1.30 mm; ML = ±2.90 mm; DV = −4.20 mm).

For silencing downstream neurons of pBLA, a 1:1 volume mixture of AAV‐hSyn‐Cre and AAV‐DIO‐DTA (150 nl per side) was bilaterally injected into the IC at 2 sites along anterior‐posterior axis (AP = +1.00 mm; ML = ±3.50 mm; DV = −3.80 mm, and AP = −0.20 mm; ML = ±4.00 mm; DV = −4.00 mm), BNST (AP = +0.25 mm; ML = ±1.0 mm; DV = −4.05 mm), or CeA (AP = −1.30 mm; ML = ±2.90 mm; DV = −4.50 mm).

For retrograde trans‐synaptic tracing using RV, Retro‐AAV‐hSyn‐Cre (60 nl) was injected into the unilateral BNST (AP = +0.25 mm; ML = 1.0 mm; DV = −4.05 mm), and a 1:1 volume mixture of AAV‐DIO‐TVA‐EGFP and AAV‐DIO‐RVG (80 nl) was injected into the unilateral pBLA (AP = −2.40 mm; ML = 3.30 mm; DV = −4.95 mm). After 14 days, RV‐EnvA‐ΔG‐dsRed (70 nl) was injected into the same site in the pBLA (AP = −2.40 mm; ML = 3.30 mm; DV = −4.95 mm). The mice were then perfused 7 days after infusion of RV‐EnvA‐ΔG‐dsRed.

For ablation of the GABAergic neurons of BNST or CeA together with pBLA glutamatergic neurons in GAD2‐Cre mice, AAV‐DIO‐DTA (150 nl per side) was injected into the bilateral BNST (AP = +0.25 mm; ML = ±1.0 mm; DV = −4.05 mm) or CeA (AP = −1.30 mm; ML = ±2.90 mm; DV = −4.50 mm). A 1:1 volume mixture of AAV‐CaMKIIα‐Flp and AAV‐fDIO‐DTA (150 nl per side) was injected into the bilateral pBLA (AP = −2.40 mm; ML = ±3.30 mm; DV = −4.95 mm).

For optogenetic activation of the vCA1‐pBLA circuit, AAV‐CaMKIIα‐ChR2‐mCherry (80 nl per side) was injected into the bilateral vCA1 (AP = −3.50 mm; ML = ±3.40 mm; DV = −3.80 mm), and optical fibers were bilaterally implanted above the pBLA (AP = −2.40 mm; ML = ±3.30 mm; DV = −4.65 mm).

For optogenetic activation of the aPVT‐pBLA circuit in vGlut2‐Cre mice, AAV‐DIO‐ChR2‐EGFP (80nl) was injected into the aPVT (AP = −0.40 mm; ML = 0.9 mm; DV = −3.50 mm, with a 15° angle toward the midline), and optical fibers were bilaterally implanted above the pBLA (AP = −2.40 mm; ML = ±3.30 mm; DV = −4.65 mm).

For optogenetic activation of the Pir‐pBLA circuit, AAV‐CaMKIIα‐ChR2‐mCherry (80 nl per side) was injected into the bilateral Pir (AP = −0.80 mm; ML = ±3.60 mm; DV = −5.30 mm), and optical fibers were bilaterally implanted above the pBLA (AP = −2.40 mm; ML = ±3.30 mm; DV = −4.65 mm).

For ablation of pBLA glutamatergic neurons, a 1:1 volume mixture of AAV‐CaMKIIα‐Cre and AAV‐DIO‐DTA (150 nl per side) was bilaterally injected into the pBLA (AP = −2.40 mm; ML = ±3.30 mm; DV = −4.95 mm).

For chemogenetic inhibition of pBLA glutamatergic neurons, AAV‐CaMKIIα‐hM4Di‐mCherry (150 nl per side) was bilaterally injected into the pBLA (AP = −2.40 mm; ML = ±3.30 mm; DV = −4.95 mm).

### Optogenetic Stimulation, Genetic Ablation, and Chemogenetic Inhibition Experiments

For optogenetic activation, the implanted optical fibers (200‐µm diameter, NA 0.37, Inper, China) were connected to a laser generator via optic fiber sleeves. The blue light (473 nm, 3–5 mW, 5‐ms pulse width, 20 Hz) was delivered to selectively activate the soma and circuits under the control of an optogenetic system (Inper, China).

For genetic ablation experiments, NeuN (neuron‐specific nuclear protein) staining was used to check the lesion area after the experiments.

For chemogenetic inhibition of pBLA glutamatergic neurons in the intrahippocampal kainic acid (KA)‐induced acute seizure model, clozapine N‐oxide (CNO) (5 mg kg^−1^, Sigma, C0832, USA) was injected intraperitoneally. 0.5 h after CNO injection, KA (0.25 µg in 500 nl saline, Abcam, ab120100, UK) was administered into the right CA1 region (AP = −2.10 mm; ML = −1.50 mm; DV = −1.60 mm) to induce acute seizures.

### EEG Recording and Analysis

The EEG signals were collected from the surface of the mouse skull. Mice were anesthetized and implanted with EEG electrodes. Three microscrews are screwed into the surface of the skull to serve as the positive electrode, negative electrode, and reference electrode. The coordinates for the insertion locations of the positive, negative, and reference electrodes are as follows: right frontal area (AP = +2.0 mm; ML = −1.5 mm), left parietal area (AP = −2.7 mm; ML = +2.4 mm), and right cerebellar area (AP = −5.6 mm; ML = −1.0 mm). The positive and negative electrodes, referred to as active electrodes, measure the corresponding surface potentials of the skull, recorded as *V*_positive_ and *V*_negative_, respectively. The reference electrode provides a relatively stable potential reference to compare the voltage difference between the positive and negative electrodes, recorded as *V*_reference_. The collected EEG data is calculated as: (*V*_positive_ − *V*_reference_) − (*V*_negative_ − *V*_reference_). EEG signals were recorded at a sampling frequency of 1000 Hz using a PowerLab system (AD instruments, Australia). The EEG analysis was performed using LabChart Pro software (v8.0, AD instruments, Australia), with brain waves divided into five different frequencies: delta (δ) wave (0.5–4 Hz), theta (θ) wave (4–8 Hz), alpha (α) wave (8–12 Hz), beta (β) wave (12–30 Hz), and gamma (γ) wave (30–500 Hz).

EEG analysis in optogenetic activation experiments: The analysis of EEG signals induced by optogenetic activation was divided into two scenarios: 1) If optogenetic activation induces epileptiform discharges, the analysis was performed on the EEG signals during the entire epileptiform discharge period. 2) If optogenetic activation does not induce epileptiform discharges, the analysis was performed on the EEG signals during the light stimulation period.

EEG analysis in the intrahippocampal KA‐induced acute seizure model: KA (0.25 µg in 500 nl saline, Abcam, ab120100, UK) was injected into the right CA1 region (AP = −2.10 mm; ML = −1.50 mm; DV = −1.60 mm) through a cannula at a speed of 200 nl min^−1^ with a 10‐µl microliter syringe (Gaoge Industry and Trade Co., Ltd., China). Then, EEG signals and videos were continuously recorded for 60 min in freely moving mice to measure seizures. The analysis of EEG signals was divided into two scenarios: 1) If the mouse does not die within the recorded 60 min, the EEG signals during the entire 60 min period were analyzed; 2) If the mouse dies within the recorded 60 min, the EEG signals from the start of the recording until the time of death were analyzed.

EEG analysis in the chronic TLE model: The chronic TLE model was established by administering an intrahippocampal injection of KA, following the methodology outlined in previous study.^[^ ^52^ ^]^ Specifically, KA (0.25 µg in 500 nl saline, Abcam, ab120100, UK) was injected into the right CA1 region (AP = −2.10 mm; ML = −1.50 mm; DV = −1.60 mm) to induce status epilepticus (SE) in mice. Subsequently, the mice were allowed to recover from anesthesia naturally. Seven weeks post‐KA injection, EEG recordings were performed continuously for 5 days (8 h per day) in freely moving mice. Most recorded discharges consisted of spontaneous high‐voltage spikes, devoid of noticeable behavioral alterations. During analysis, spontaneous high‐voltage spikes exceeding 0.5 mV in amplitude, measured from baseline to positive peaks, were detected and examined.

### The Assessment of Seizure Behaviors

At the beginning of the study, to determine whether the behaviors induced by optogenetic activation of the pBLA glutamatergic neurons were epileptic seizures, EEG monitoring was conducted alongside recording accompanying symptoms of epilepsy, such as pupillary dilation and foaming at the mouth. These observations from multiple perspectives confirmed that the behavior induced by the pBLA glutamatergic neuron activation was indeed characterized by epileptic seizures. However, since EEG is the gold standard for determining epileptic seizures,^[^ ^53^, ^54^ ^]^ subsequent experiments in this study focused on monitoring EEG as the primary criterion for evaluating seizures, without further attention to accompanying symptoms like pupillary dilation and foaming at the mouth.

Seizure severity was classified into six stages according to the modified Racine scale:^[^ ^55^ ^]^ 0) no abnormality, 1) facial movement, 2) head nodding, 3) unilateral forelimb clonus, 4) bilateral forelimb clonus and rearing, 5) falling, and 6) death. A trained observer, unaware of the experimental groupings, scored the seizure severity.

In the intrahippocampal KA‐induced acute seizure model, generalized seizures (GSs) were identified by characteristic EEG patterns. GS discharges were marked by brief high‐amplitude EEG signals followed by flattened EEG signals (postictal generalized EEG suppression, PGES) (Figure 9C), a pattern typically occurring during severe seizures.^[^ ^56^, ^57^, ^58^ ^]^ All GS events in each mouse were rated according to the seizure rating scale over a 60 min monitoring period, and the average of these ratings was taken as the GS stage for that mouse.

### Pupil Size Measurement

Mice were head fixed to measure pupil size, recorded using a camera under infrared lighting. The whole measurement process for each mouse included three stages: pre (10 s‐light off), light on (10 s‐light on), and post (10 s‐light off). The recorded videos were converted to pictures (30 frames s^−1^) using Adobe Premiere Pro software (Premiere 2022, Adobe Inc., USA). One picture was selected each second and 30 pictures from the whole measurement process were obtained for pupil size analysis. The area value of pupil size in every picture was calculated using ImageJ software (v2.15.1, NIH, USA). The mean area value of pupil size of 10 pictures in the pre stage was the normalized number. The area value of pupil size of 30 pictures across the whole measurement process was divided by the normalized number to obtain a curve plot of normalized pupil size.

### Recording Foaming at the Mouth

Mice were held by one hand with cotton gloves, and videos were recorded using an Apple iPhone 12 Pro. The whole recording process of each mouse included three stages: pre (10 s‐light off), light on (30 s), and post (10 s‐light off). The recorded videos were converted by Adobe Premiere Pro (Premiere 2022, Adobe Inc., USA) to obtain pictures of foaming at the mouth.

### In Vivo Fiber Photometry and EEG Recordings in Intrahippocampal KA‐Induced Acute Seizure Model

After GCaMP6s virus expression for 14 days, mice were implanted with cannulas for KA injection, optical fibers for calcium signal recordings, and EEG electrodes for EEG signal recordings, as described above. After 7 days of recovery, KA (0.25 ug in 500 nl saline, Abcam, ab120100, UK) was injected into the CA1 through the cannula at a speed of 200 nl min^−1^ using a 10 µl microliter syringe (Gaoge Industrial and Trading Co. Ltd., China). It was then recorded calcium signals using a fiber photometry system with two excitation wavelengths: 405‐nm (comparison for GCaMP signal; calcium independent) and 465‐nm (GCaMP signal; calcium dependent) (Inper, China), recorded EEG signals using the PowerLab system, and recorded the computer screen using Bandicam software (v5.0, Bandicam Company, South Korea) for 60 min. For fiber photometry, the power, sampling frequency, and sampling time of 465‐nm excitation light at the tip of the optical fiber were: 10 µW, 50 Hz, and 5 ms, respectively; the power, sampling frequency, and sampling time of 405‐nm excitation light at the tip of optical fiber were: 2 µW, 50 Hz, and 5 ms, respectively. The baseline was defined by the signals collected during the 5‐second interval following the start of recording, with *F0* representing the averaged baseline fluorescence. Fluorescence changes were quantified by *∆F/F*  =  (*F − F0*) / *F0*. Calcium signal data were analyzed using Inper software (v1.0, Inper, China).

### Histological Analysis

Mice were deeply anesthetized with sodium pentobarbital (50 mg kg^−1^, i.p.) and perfused with ≈30 ml of saline (0.9%, w/v), followed by ≈30 ml of ice‐cold 4% paraformaldehyde (PFA) in phosphate‐buffered saline (PBS). Brains were carefully extracted from the skull and postfixed in 4% PFA for 24 h, then dehydrated with 30% sucrose in PBS at 4 °C. The brains were sectioned into 50‐µm coronal slices using a freezing microtome (CM1950, Leica, Germany). Slices were collected and stored in a freezing buffer (PBS: Ethylene glycol: Glycerin = 5: 3: 2) at −20 °C until immunofluorescent processing.

For immunofluorescent staining, brain slices were washed in PBS three times (10 min each time) and incubated with blocking buffer containing 4% bovine serum albumin (Amresco, 0332–500G, USA) dissolved in 0.4% PBST (0.4% Triton X‐100 in PBS) for 1 h at room temperature. Slices were then incubated with primary antibodies diluted in blocking buffer overnight at 4 °C. After incubation, the slices were washed in PBS three times (10 min each time), then transferred into blocking buffer containing a fluorescent dye‐conjugated secondary antibody for 1 h at room temperature. Following three washes (10 min each time) with PBS, the slices were incubated with 4′,6‐diamidino‐2‐phenylindole (DAPI, 1:1 000, Sigma–Aldrich, MBD0015, USA) for 5 min, then washed and mounted under coverslips with Fluoromount aqueous mounting medium (Sigma–Aldrich, F4680, USA).

The primary antibody: NeuN (rabbit, 1:1 000, Cell Signaling Technology, 24307S, USA).

The secondary antibodies: Alexa Flour 488 goat anti‐rabbit IgG (1:500, Yeasen Biotechnology, 33106ES60, China), Alexa Fluor 594 goat anti‐rabbit IgG (1:500, Yeasen Biotechnology, 33126ES60, China).

Slides were imaged at 10× objective with an Olympus VS120 (Olympus, Japan) virtual slide microscope system and further processed by Olympus OlyVIA software (v3.12, Olympus, Japan).

### Fluorescence In Situ Hybridization

Fluorescence in situ hybridization (FISH) was performed using a RNAscope Fluorescent Multiplex Kit (ACD Bio, 323 100, USA). Coronal brain slices (20 µm) were prepared as described above. The brain slices were dried for 20 min at 60 °C in a baking oven, with hydrophobic circles drawn around them. All subsequent steps were performed in a 40 °C incubator. Proteins were digested with protease III solution for 30 min, and the probes were heated in the same incubator. After two washes with RNAscope wash buffer, the probes were applied to the slices for 2 h in the 40 °C incubator. Following another two washes with RNAscope wash buffer, the slices underwent reaction steps with AMP1‐3 and HRP1‐3, depending on the chosen color combination. The probes used included *Slc17a7* (ACD Bio, 501101‐C1, USA), and *Slc32a1* (ACD Bio, 319191‐C2, USA).

Slices were imaged using a 10x or 40x objective with an Olympus FV3000 microscope system (Olympus, Japan) and further processed with ImageJ software (v2.15.1, NIH, USA).

### The Blinding Strategy used for Cell Counting

Allocation Stage: The group allocation was conducted by Experimenter 1 who was not involved in the subsequent experimental procedures or assessments; Conduct of the Experiment: During cell counting, Experimenter 2 was blinded to the group allocation; Outcome Assessment: Experimenter 3, responsible for assessing outcomes, was also blinded to the group allocation, thus reducing bias in the data collection process; Data Analysis: During data analysis, blinding was maintained as much as possible. The data were coded, and the group labels were not revealed until all analyses were completed.

### Quantification of Presynaptic Inputs using Monosynaptic Retrograde Rabies Virus (RV) Tracing

Brain slice collection and region assignments were performed according to the atlas “The Mouse Brain in Stereotaxic Coordinates (Fourth Edition)”.^[^ ^59^ ^]^ Every 50 µm section from AP +2.65 mm to AP ‐7.55 mm was collected and imaged using an Olympus VS‐120 Virtual Slide Scanning Microscope with OlyVIA software (v3.12, Olympus, Japan), maintaining identical magnification and exposure time. Neurons with dsRed‐positive cell bodies were counted manually. The injection was unilateral (left side), and cell quantification was bilateral. The percentage of total presynaptic inputs from a given brain region was calculated by dividing the number of presynaptic neurons by the total number of presynaptic neurons registered to the atlas.

### *Ex Vivo* Electrophysiology

For slice physiology combined with optogenetics, a mixture of AAV‐CaMKIIα‐mCherry and Antero‐AAV2/1‐hSyn‐Cre was injected into the pBLA, and AAV‐DIO‐ChR2‐EGFP was injected into the BNST or CeA of 8‐week‐old C57BL/6J mice. After one month, acute 300‐µm coronal slices were prepared using a Leica VT1000S vibroslice (Leica, VT1200, Germany) in an ice‐cold cutting solution, with a slicing speed of 0.1 mm s^−1^. The slices were initially incubated in a mixture of 20% cutting solution and 80% artificial cerebrospinal fluid (ACSF) at 34 °C for 15 min, then transferred to ACSF to recover at room temperature for 1 h before recording. The cutting solution contained 230 mM sucrose, 2.5 mM KCl, 10 mM MgSO_4_, 0.5 mM CaCl_2_.2H_2_O, 1.25 mM NaH_2_PO_4_, 26 mM NaHCO_3_, 10 mM glucose, and 1.5 mM sodium pyruvate. The ACSF contained 126 mM NaCl, 2.5 mM KCl, 1.25 mM NaH_2_PO_4_, 26 mM NaHCO_3_, 2 mM CaCl_2_.2H_2_O, 2 mM MgCl_2_.6H_2_O, and 10 mM glucose. All solutions were saturated with 95% O_2_ and 5% CO_2_.

For recordings, slices were transferred to a recording chamber perfused with ACSF containing 126 mM NaCl, 2.5 mM KCl, 1.25 mM NaH_2_PO_4_, 26 mM NaHCO_3_, 2 mM CaCl_2_.2H_2_O, 2 mM MgCl_2_.6H_2_O, and 10 mM glucose, saturated with 95% O_2_ and 5% CO_2_. Whole‐cell patch‐clamp recordings were performed on pBLA cells with a MultiClamp 700B amplifier (2‐kHz low‐pass filter, 10 kHz digitization, Molecular Devices, USA) and a 1440A interface (Molecular Devices, USA) using pClamp v10.4 (Molecular Devices, USA). A 473‐nm blue light was delivered by an LED (Polygon400, Mightex, Canada) directed through the objective with a light intensity of ≈2 mW. Fluorescent cells in the pBLA region were visualized under an Eclipse FN1 microscope (Nikon, Japan) equipped with a 40x water‐immersion lens and illuminated with a mercury lamp.

Light‐evoked postsynaptic currents were recorded using pipettes (3–5 MΩ) filled with an intracellular solution containing 140 mM Cs‐methanesulfonate, 5 mM NaCl, 1 mM MgCl_2_.6H_2_O, 10 mM HEPES, 0.2 mM EGTA, 2 mM Mg‐ATP, and 0.5 mM Na‐GTP (pH 7.3). Recordings were conducted in the voltage‐clamp mode, holding the membrane potentials of neurons at 0 mV to record eIPSCs and at ‐70 mV to record eEPSCs. TTX (1 µM, Alomone Labs, T‐550, Israel) was used to block voltage‐gated sodium channels, followed by 4‐AP (100 µM, Sigma–Aldrich, A78403, USA) to confirm monosynaptic postsynaptic currents following light activation. Picrotoxin (100 µM, Tocris Bioscience, 1128, UK) was bath‐applied to validate whether the recorded eIPSCs were mediated by GABA receptors. Data were analyzed using Clampfit software (v10.4, Molecular Devices, USA).

### Statistical Analysis

No animals, experimental units, or data points were excluded from the analysis in any experimental group, and all collected data were included in the final analysis. The main experiments were successfully replicated in the laboratory across at least two cohorts, with representative images in the figures obtained from at least three mice. The data represent biological replicates. Sample sizes in our study were not predetermined using any statistical methods but were comparable to those in previous publications.^[^ ^60^, ^61^, ^62^, ^63^ ^]^ All mice were randomly assigned to either the control or experimental group. Data collection and processing were conducted in a randomized or counterbalanced manner, and experimenters were blinded to the treatments. Data analysis was carried out without knowledge of the treatment conditions.

All data were expressed as the mean ± standard error of the mean (SEM). Statistical analyses were performed using GraphPad Prism (v8.0, GraphPad Software, Inc., USA). For paired sample experiments, Wilcoxon test and Friedman test were used. For non‐paired sample experiments, comparisons between two groups were performed using the Mann‐Whitney U‐test, Chi‐square test, Log‐rank test or two‐tailed unpaired t‐test, while comparisons among multiple groups were done using one‐way analysis of variance (ANOVA) followed by Tukey's test or Fisher's least significant difference (LSD) test. The significance threshold was held at α = 0.05 (ns, not significant, *P* > 0.05; *P < 0.05; **P < 0.01; ***P < 0.001; ****P < 0.0001).

## Conflict of Interest

The authors declare no conflict of interest.

## Author Contributions

Y.‐H.S., X.‐M.L., and J.C. initiated and designed the research and wrote the manuscript. Y.‐H.S. conducted the experiments and analyzed the results with an assistant from B.‐W.H., L.‐H.T., T.‐X.W., and L.L. assisted with the histology. B.Y., H.W., Q.W., S.‐X.C., and H.L. contributed to the discussion of the results. X.‐M.L., J.C., S.‐X.C., and H.L. acquired the funding. X.‐M.L. and J.C. supervised the entire project.

## Ethical Statement

Ethics approval for this study was obtained from the Laboratory Animal Welfare and Ethics Committee of Zhejiang University (authorization: ZJU20220384).

## Supporting information



Supporting Information

Supplemental Movie 1

Supplemental Movie 2

Supplemental Movie 3

Supplemental Table 1

## Data Availability

The data that support the findings of this study are available from the corresponding author upon reasonable request.

## References

[1] S. L. Moshe , E. Perucca , P. Ryvlin , T. Tomson , Lancet 2015, 385, 884. 25260236 10.1016/S0140-6736(14)60456-6

[2] R. S. Fisher , C. Acevedo , A. Arzimanoglou , A. Bogacz , J. H. Cross , C. E. Elger , J. Engel Jr. , L. Forsgren , J. A. French , M. Glynn , D. C. Hesdorffer , B. I. Lee , G. W. Mathern , S. L. Moshe , E. Perucca , I. E. Scheffer , T. Tomson , M. Watanabe , S. Wiebe , Epilepsia 2014, 55, 475. 24730690 10.1111/epi.12550

[3] M. R. Pascual , Semin Ultrasound CT MR 2007, 28, 416. 18074998 10.1053/j.sult.2007.09.004

[4] A. A. Asadi‐Pooya , L. Bartolini , Int. J. Neurosci. 2020, 130, 1151. 32053411 10.1080/00207454.2020.1730370

[5] P. Bencurova , H. Laakso , R. A. Salo , E. Paasonen , E. Manninen , J. Paasonen , S. Michaeli , S. Mangia , M. Bares , M. Brazdil , H. Kubova , O. Grohn , Neurobiol. Dis. 2022, 162, 105566. 34838665 10.1016/j.nbd.2021.105566 PMC8845085

[6] J. F. Tellez‐Zenteno , L. Hernandez‐Ronquillo , Epilepsy Res. Treat 2012, 2012, 630853. 22957234 10.1155/2012/630853 PMC3420432

[7] L. El‐Hassar , M. Milh , F. Wendling , N. Ferrand , M. Esclapez , C. Bernard , J. Physiol. 2007, 578, 193. 17008374 10.1113/jphysiol.2006.119297 PMC2075107

[8] K. Lukasiuk , M. Dabrowski , A. Adach , A. Pitkanen , Prog. Brain Res. 2006, 158, 223. 17027699 10.1016/S0079-6123(06)58011-2

[9] Y. H. Raol , I. V. Lund , S. Bandyopadhyay , G. Zhang , D. S. Roberts , J. H. Wolfe , S. J. Russek , A. R. Brooks‐Kayal , J. Neurosci. 2006, 26, 11342. 17079662 10.1523/JNEUROSCI.3329-06.2006 PMC6674546

[10] M. Navidhamidi , M. Ghasemi , N. Mehranfard , Rev. Neurosci. 2017, 28, 307. 28099137 10.1515/revneuro-2016-0059

[11] M. Wenzel , G. Huberfeld , D. B. Grayden , M. de Curtis , A. J. Trevelyan , Epilepsia 2023, 64, S37. 37183507 10.1111/epi.17650

[12] P. Mohapel , C. Dufresne , M. E. Kelly , D. C. McIntyre , Epilepsy Research 1996, 23, 179. 8739121 10.1016/0920-1211(95)00084-4

[13] E. W. Kairiss , R. J. Racine , G. K. Smith , Brain Res. 1984, 322, 101. 6518361 10.1016/0006-8993(84)91185-5

[14] V. Aroniadou‐Anderjaska , B. Fritsch , F. Qashu , M. F. M. Braga , Epilepsy Research 2008, 78, 102. 18226499 10.1016/j.eplepsyres.2007.11.011 PMC2272535

[15] F. Cendes , F. Leproux , D. Melanson , R. Ethier , A. Evans , T. Peters , F. Andermann , J. Comp. Assist. Tomograp. 1993, 17, 206. 10.1097/00004728-199303000-00008 8454746

[16] A. Saukkonen , R. Kälviäinen , K. Partanen , P. Vainio , P. Riekkinen , A. Pitkänen , NeuroReport 1994, 6, 219. 7703420 10.1097/00001756-199412300-00055

[17] A. Pitka¨nen , J. Tuunanen , R. Ka¨lvia¨inen , K. Partanen , T. Salmenpera , Epilepsy Research 1998, 32, 233. 9761324 10.1016/s0920-1211(98)00055-2

[18] S. Graebenitz , O. Kedo , E. J. Speckmann , A. Gorji , H. Panneck , V. Hans , N. Palomero‐Gallagher , A. Schleicher , K. Zilles , H. C. Pape , Brain 2011, 134, 2929. 21893592 10.1093/brain/awr202

[19] J. Schramm , Epilepsia 2008, 49, 1296. 18410360 10.1111/j.1528-1167.2008.01604.x

[20] W. Feindel , T. Rasmussen , Can. J. Neurol. Sci. 1991, 18, 603. 1777879 10.1017/s0317167100032807

[21] R. Jooma , H. S. Yeh , M. D. Privitera , D. Rigrish , Acta Neurochir. 1995, 133, 44. 8561035 10.1007/BF01404946

[22] E. Sah , M. Lopez de Armentia , A. Power , Physiol. Rev. 2003, 83, 803. 12843409 10.1152/physrev.00002.2003

[23] G. D. P. Larry , W. Swanson , Trends Neurosci. 1998, 21, 323. 9720596 10.1016/s0166-2236(98)01265-x

[24] P. H. Janak , K. M. Tye , Nature 2015, 517, 284. 25592533 10.1038/nature14188 PMC4565157

[25] A. M. Kanner , M. M. Bicchi , JAMA, J. Am. Med. Assoc. 2022, 327, 1269. 10.1001/jama.2022.3880 35380580

[26] C. Brittain , G. Ambegaonkar , Arch. Dis. Child Educ. Pract. Ed. 2018, 103, 302. 28939550 10.1136/archdischild-2017-313314

[27] J. Shah , H. Zhai , D. Fuerst , C. Watson , Epilepsia 2006, 47, 644. 16529634 10.1111/j.1528-1167.2006.00480.x

[28] C. T. Primiani , K. S. Husari , R. Mathur , R. G. Geocadin , J. Neurocrit. Care 2022, 15, 136.

[29] A. M. Douglass , J. M. Resch , J. C. Madara , H. Kucukdereli , O. Yizhar , A. Grama , M. Yamagata , Z. Yang , B. B. Lowell , Nature 2023, 620, 154. 37495689 10.1038/s41586-023-06358-0 PMC11168300

[30] A. Cukiert , C. Forster , M. S. D. Andrioli , L. Frayman , Arq Neuro‐Psiquiat 1998, 56, 126. 10.1590/s0004-282x1998000100022 9686134

[31] X. Zhang , G. Zhang , T. Yu , C. Xu , J. Zhu , X. Yan , K. Ma , R. Gao , Medicine 2022, 101, e30114. 35984139 10.1097/MD.0000000000030114 PMC9387976

[32] J. F. Poulin , D. Arbour , S. Laforest , G. Drolet , Prog Neuropsychopharmacol Biol Psychiatry 2009, 33, 1356. 19583989 10.1016/j.pnpbp.2009.06.021

[33] A. Q. Nguyen , J. A. Dela Cruz , Y. Sun , T. C. Holmes , X. Xu , J Comp Neurol 2016, 524, 2379. 26718312 10.1002/cne.23954 PMC5359980

[34] I. Ehrlich , Y. Humeau , F. Grenier , S. Ciocchi , C. Herry , A. Luthi , Neuron 2009, 62, 757. 19555645 10.1016/j.neuron.2009.05.026

[35] J. T. Paz , J. R. Huguenard , Nat. Neurosci. 2015, 18, 351. 25710837 10.1038/nn.3950 PMC4561622

[36] C. Tai , Y. Abe , R. E. Westenbroek , T. Scheuer , W. A. Catterall , Proc. Natl. Acad. Sci. USA 2014, 111, E3139. 25024183 10.1073/pnas.1411131111 PMC4121787

[37] I. Cobos , M. E. Calcagnotto , A. J. Vilaythong , M. T. Thwin , J. L. Noebels , S. C. Baraban , J. L. R. Rubenstein , Nat. Neurosci. 2005, 8, 1059. 16007083 10.1038/nn1499

[38] J. R. Huguenard , D. A. McCormick , Trends Neurosci. 2007, 30, 350. 17544519 10.1016/j.tins.2007.05.007

[39] K. Morimoto , M. Fahnestock , R. J. Racine , Prog Neurobiol 2004, 73, 1. 15193778 10.1016/j.pneurobio.2004.03.009

[40] B. A. Strange , M. P. Witter , E. S. Lein , E. I. Moser , Nat. Rev. Neurosci. 2014, 15, 655. 25234264 10.1038/nrn3785

[41] T. L. Babb , W. J. Brown , J. Pretorius , C. Davenport , J. P. Lieb , P. H. Crandall , Epilepsia 1984, 25, 729. 6510381 10.1111/j.1528-1157.1984.tb03484.x

[42] A. M. Dam , Epilepsia 1980, 21, 617. 6777154 10.1111/j.1528-1157.1980.tb04315.x

[43] W. M. OConnor , L. Masukawa , A. Freese , M. R. Sperling , J. A. French , M. J. OConnor , Epilepsia 1996, 37, 440. 8617172 10.1111/j.1528-1157.1996.tb00589.x

[44] M. Thom , Neuropathol. Appl. Neurobiol. 2014, 40, 520. 24762203 10.1111/nan.12150 PMC4265206

[45] D. N. Vaughan , G. D. Jackson , Front. Neurol. 2014, 5, 259. 25538678 10.3389/fneur.2014.00259 PMC4259123

[46] Y. Shima , H. Skibbe , Y. Sasagawa , N. Fujimori , Y. Iwayama , A. Isomura‐Matoba , M. Yano , T. Ichikawa , I. Nikaido , N. Hattori , T. Kato , Cell Rep. 2023, 42, 113309. 37862168 10.1016/j.celrep.2023.113309

[47] H. E. Scharfman , N. J. MacLusky , Neurobiol. Dis. 2014, 72, 180. 25058745 10.1016/j.nbd.2014.07.004 PMC4252793

[48] J. Christensen , M. J. Kjeldsen , H. Andersen , M. L. Friis , P. Sidenius , Epilepsia 2005, 46, 956. 15946339 10.1111/j.1528-1167.2005.51204.x

[49] I. Savic , Experimen. Neurol. 2014, 259, 38. 10.1016/j.expneurol.2014.04.009 24747359

[50] O. Baud , S. Desgent , S. Duss , N. T. Sanon , P. Lema , M. Lévesque , D. Hébert , R.‐M. Rébillard , K. Bibeau , M. Brochu , L. Carmant , PLoS One 2012, 7, e42622. 22880055 10.1371/journal.pone.0042622 PMC3411822

[51] M. Salzberg , G. Kumar , L. Supit , N. C. Jones , M. J. Morris , S. Rees , T. J. O'Brien , Epilepsia 2007, 48, 2079. 17999745 10.1111/j.1528-1167.2007.01246.x

[52] N. Sada , S. Lee , T. Katsu , T. Otsuki , T. Inoue , Science 2015, 347, 1362. 25792327 10.1126/science.aaa1299

[53] A. Goenka , A. Boro , E. Yozawitz , Epileptic Disorders 2017, 19, 299. 28721936 10.1684/epd.2017.0921

[54] L. J. Hirsch , R. P. Brenner , F. W. Drislane , E. So , P. W. Kaplan , K. G. Jordan , S. T. Herman , S. M. LaRoche , B. Young , T. P. Bleck , M. L. Scheuer , R. G. Emerson , J. Clin. Neurophysiol. 2005, 22, 128. 15805813 10.1097/01.wnp.0000158701.89576.4c

[55] R. J. Racine , Electroencephalogr. Clin. Neurophysiol. 1972, 32, 281. 4110397 10.1016/0013-4694(72)90177-0

[56] R. Guerrini , C. Marini , C. Barba , Clinical Neurophysiology: Diseases and Disorders, Elsevier, Amsterdam, Netherlands 2019.

[57] J. Xu , B. Jin , J. Yan , J. Wang , J. Hu , Z. Wang , Z. Chen , M. Ding , S. Chen , S. Wang , Clin. Neurophysiol. 2016, 127, 2078. 26851982 10.1016/j.clinph.2015.10.064

[58] J. Y. Kang , A. H. Rabiei , L. Myint , M. Nei , Seizure 2017, 48, 28. 28380395 10.1016/j.seizure.2017.03.017

[59] K. B. J. Franklin , G. Paxinos , The Mouse Brain in Stereotaxic Coordinates, Elsevier, Amsterdam, Netherlands 2013.

[60] C.‐J. Shen , D. Zheng , K.‐X. Li , J.‐M. Yang , H.‐Q. Pan , X.‐D. Yu , J.‐Y. Fu , Y. Zhu , Q.‐X. Sun , M.‐Y. Tang , Y. Zhang , P. Sun , Y. Xie , S. Duan , H. Hu , X.‐M. Li , Nat. Med. 2019, 25, 337. 30643290 10.1038/s41591-018-0299-9

[61] V. A. Kveim , L. Salm , T. Ulmer , M. Lahr , S. Kandler , F. Imhof , F. Donato , Science 2024, 385, eadk0997. 39146420 10.1126/science.adk0997

[62] F. Fei , X. Wang , C. Xu , J. Shi , Y. Gong , H. Cheng , N. Lai , Y. Ruan , Y. Ding , S. Wang , Z. Chen , Y. Wang , Nat. Commun. 2022, 13, 5010. 36008421 10.1038/s41467-022-32742-x PMC9411516

[63] B. Chen , C. Xu , Y. Wang , W. Lin , Y. Wang , L. Chen , H. Cheng , L. Xu , T. Hu , J. Zhao , P. Dong , Y. Guo , S. Zhang , S. Wang , Y. Zhou , W. Hu , S. Duan , Z. Chen , Nat. Commun. 2020, 11, 923. 32066723 10.1038/s41467-020-14648-8 PMC7026152

