# Supplementary material for: Posterior Basolateral Amygdala is a Critical Amygdaloid Area for Temporal Lobe Epilepsy
Source: Adv Sci (Weinh). 2024 Oct 30;11(48):2407525. doi: 10.1002/advs.202407525 (PMC11672268; doi:10.1002/advs.202407525)
Supplement: Supplementary file 1 — Supporting Information [file ADVS-11-2407525-s001.docx]

# Supporting Information

Posterior basolateral amygdala is a critical amygdaloid area for temporal lobe epilepsy

*Yan-Hui Sun, Bo-Wu Hu, Li-Heng Tan, Lin Lin, Shu-Xia Cao, Tan-Xia Wu, Hao Wang, Bin Yu, Qin Wang, Hong Lian, Jiadong Chen,* and Xiao-Ming Li**

**This PDF file includes:**

Figures S1 to S11

**Other Supporting Information for this manuscript includes the following:**

Supporting Data. Statistics and Source Data

Movies S1 to S3

Movie S1. Optogenetic activation of pBLA glutamatergic neurons induces epileptiform discharges, seizures, and even death.

Movie S2. Mice with ablation of BNST neurons exhibit sporadic seizures.

Movie S3. Mice with ablation of CeA neurons exhibit sporadic seizures.


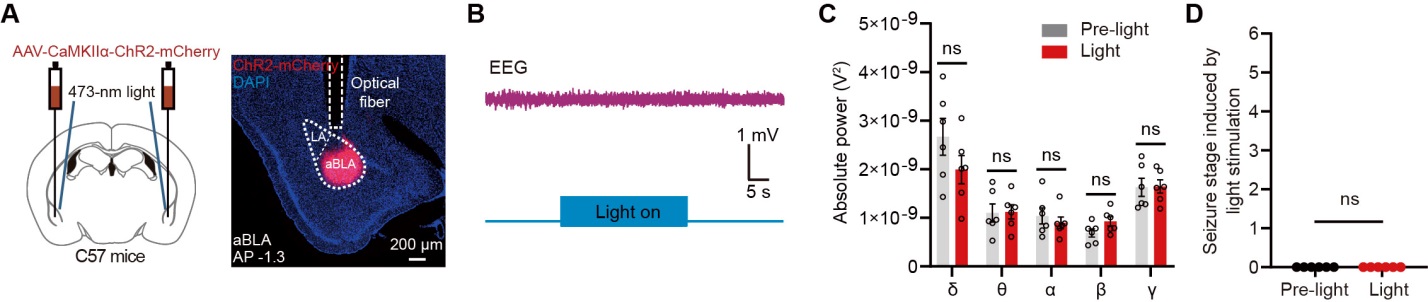


1. Figure S1. Optogenetic activation of aBLA glutamatergic neurons does not induce epileptiform discharges or seizures.

(**A**) Left panel: Schematic of optogenetic activation of aBLA glutamatergic neurons; Right panel: Expression of ChR2-mCherry in the aBLA region and the location of optical fiber (lateral amygdala, LA). (**B**) Representative EEG traces induced by optogenetic activation of aBLA glutamatergic neurons. (**C**) EEG spectral analysis before and after optogenetic activation of aBLA glutamatergic neurons (*n* = 6 mice; two-tailed paired t-test, not significant (ns), *P* > 0.05). (**D**) Seizure stage induced by optogenetic activation of aBLA glutamatergic neurons (*n* = 6 mice; Wilcoxon test, ns, *P* > 0.05). Data are presented as mean ± standard error of the mean (SEM). AAV, adeno-associated virus; AP, anterior-posterior axis; CaMKIIα, calcium/calmodulin-dependent protein kinase IIα; ChR2, channelrhodopsin-2; DAPI, 4',6-diamidino-2-phenylindole; EEG, electroencephalography; aBLA, anterior basolateral amygdala; LA, lateral amygdala.


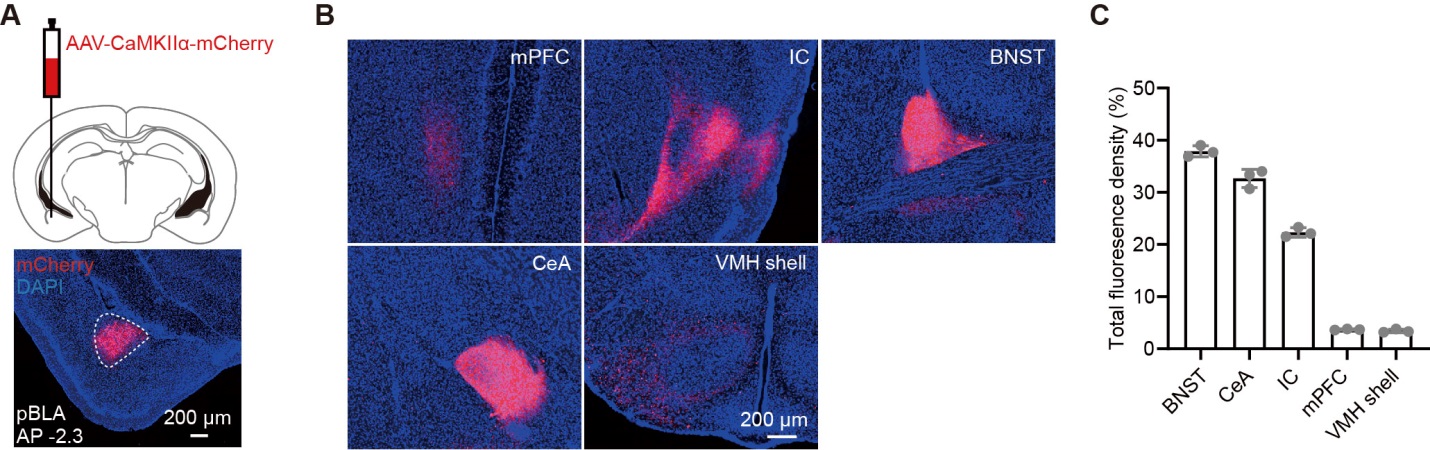


1. Figure S2. Output mapping of pBLA glutamatergic neurons.

(**A**) Schematic of viral injection and expression in pBLA. (**B**) Output mapping of pBLA glutamatergic neurons. (**C**) Quantification of relative fluorescence density of mCherry-labeled axon terminals of pBLA neurons in downstream regions (*n* = 3 mice). Data are presented as mean ± SEM. AAV, adeno-associated virus; AP, anterior-posterior axis; CaMKIIα, calcium/calmodulin-dependent protein kinase IIα; DAPI, 4',6-diamidino-2-phenylindole; pBLA, posterior basolateral amygdala; mPFC, medial prefrontal cortex; IC, insular cortex; BNST, bed nucleus of the stria terminalis; CeA, central amygdala; VMH shell, ventromedial hypothalamus shell.


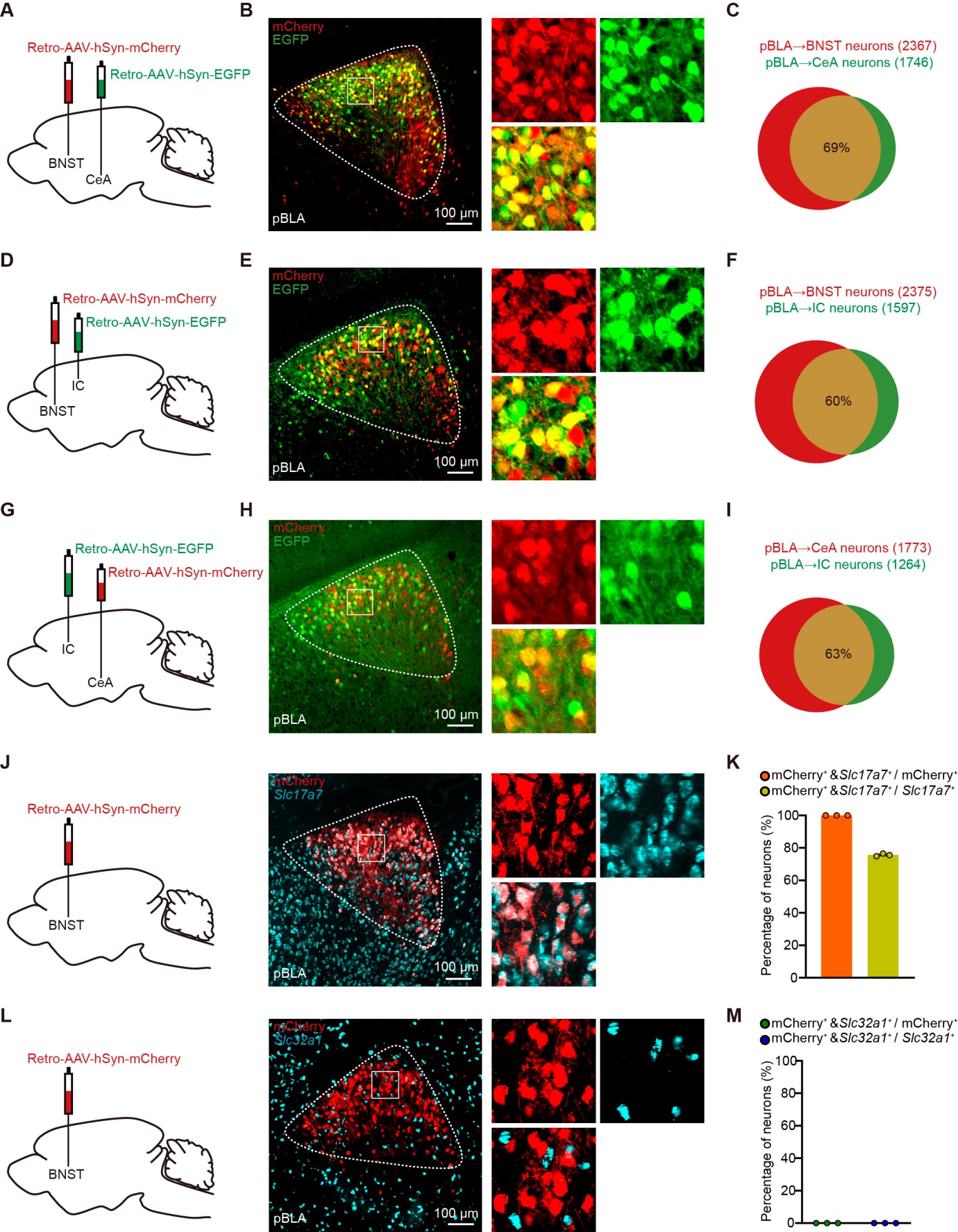


1. Figure S3. pBLA neurons collaterally project to multiple brain regions.

(**A**, **D**, and **G**) Schematic of strategy to retrogradely label pBLA neurons from two independent downstream brain regions with retro-AAV, BNST and CeA (A), IC and BNST (D), IC and CeA (G). (**B**, **E**, and **H**) Representative images showing retrograde labeling of pBLA neurons from BNST and CeA (B), IC and BNST (E), or IC and CeA (H). (**C**, **F** and **I**) Venn diagram reflecting the overlap between pBLA→BNST (2367 neurons) and pBLA→CeA neurons (1746 neurons) (C), pBLA→BNST (2375 neurons) and pBLA→IC neurons (1597 neurons) (F), or pBLA→CeA (1773 neurons) and pBLA→IC neurons (1264 neurons) (I) (*n* = 3 mice per group, 4 brain slices per mouse). (**J** and **L**) Representative images of pBLA→BNST neurons labeled with mCherry co-localized with *Slc17a7* or *Slc32a1*. (**K** and **M**) Statistical charts of pBLA→BNST neurons labeled with mCherry co-localized with *Slc17a7* or *Slc32a1*. (*n* = 3 mice per group, 2 brain slices per mouse). Data are presented as mean ± SEM. AAV, adeno-associated virus; EGFP, enhanced green fluorescent protein; pBLA, posterior basolateral amygdala; IC, insular cortex; BNST, bed nucleus of the stria terminalis; CeA, central amygdala.


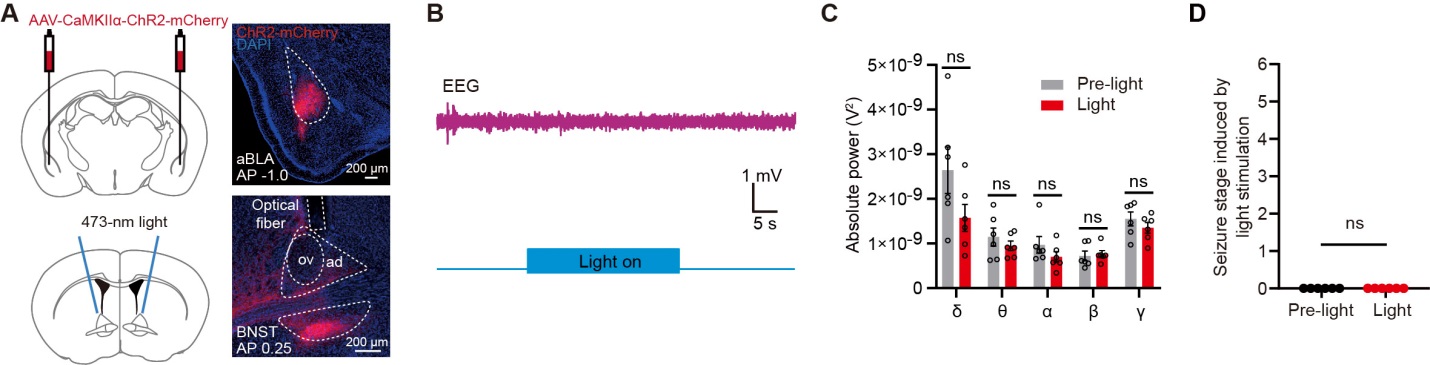


1. Figure S4. Activation of aBLA-BNST circuit does not result in epileptiform discharges or seizures.

(**A**) Left: Schematic of optogenetic activation of aBLA-BNST circuit. Right: ChR2-mCherry expression in aBLA and location of optical fiber in BNST (ov, oval; ad, anterodorsal). (**B**) Representative EEG traces induced by optogenetic activation of aBLA-BNST circuit. (**C**) EEG spectral analysis before and after optogenetic activation of aBLA-BNST circuit (*n* = 6 mice; two-tailed paired t-test, ns, *P* > 0.05). (**D**) Seizure stage induced by optogenetic activation of aBLA-BNST circuit (*n* = 6 mice; Wilcoxon test, ns, *P* > 0.05). Data are presented as mean ± SEM. AAV, adeno-associated virus; AP, anterior-posterior axis; CaMKIIα, calcium/calmodulin-dependent protein kinase IIα; ChR2, channelrhodopsin-2; DAPI, 4',6-diamidino-2-phenylindole; EEG, electroencephalography; aBLA, anterior basolateral amygdala; BNST, bed nucleus of the stria terminalis.


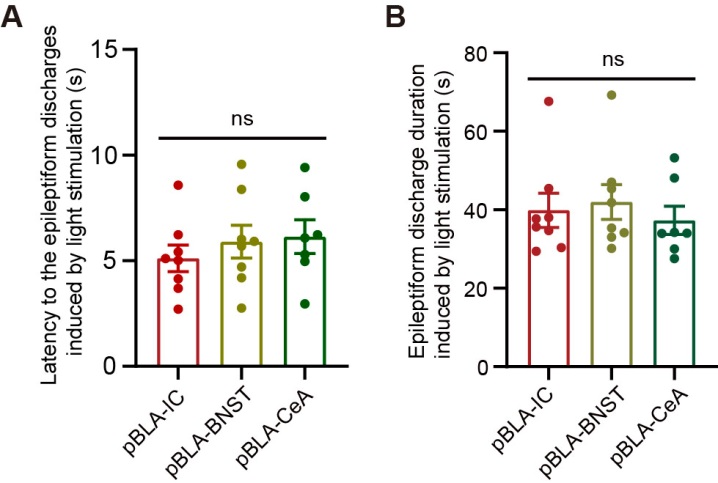


1. Figure S5. Epileptiform discharge latency and duration induced by optogenetic activation of pBLA glutamatergic axon terminals.

(**A**) Latency to the epileptiform discharges induced by optogenetic activation of pBLA glutamatergic axon terminals in the IC, BNST, or CeA (pBLA-IC: *n* = 8 mice; pBLA-BNST: *n* = 8 mice; pBLA-CeA: *n* = 7 mice; one-way ANOVA followed by Tukey’s multiple comparisons test, ns, *P* > 0.05). (**B**) The duration of epileptiform discharges induced by optogenetic activation of pBLA glutamatergic axon terminals in the IC, BNST, or CeA (pBLA-IC: *n* = 8 mice; pBLA-BNST: *n* = 8 mice; pBLA-CeA: *n* = 7 mice; one-way ANOVA followed by Tukey’s multiple comparisons test, ns, *P* > 0.05). Data are presented as mean ± SEM. pBLA, posterior basolateral amygdala; IC, insular cortex; BNST, bed nucleus of the stria terminalis; CeA, central amygdala.


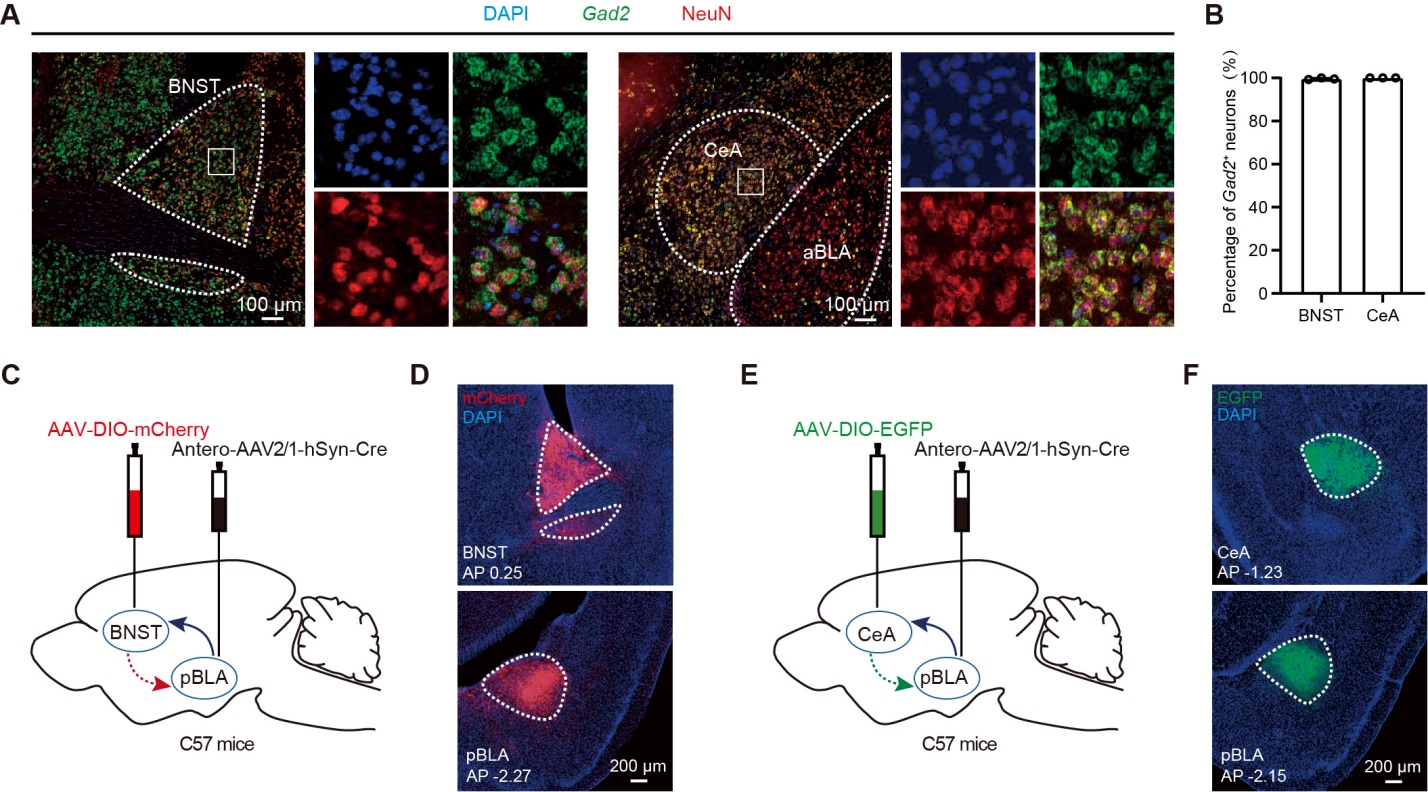


1. Figure S6. Reciprocal projections between BNST^GABA+^ neurons and pBLA neurons, as well as between CeA^GABA+^ neurons and pBLA neurons.

(A) Representative images of DAPI, *Gad2*, and NeuN distribution in the BNST and CeA regions. (B) Percentage of *Gad2*^+^ neurons in the BNST and CeA regions (BNST: *n* = 3 mice, 3-5 brain slices per mouse; CeA: *n* = 3 mice, 3-5 brain slices per mouse). (C and E) Schematic diagrams of viral injections in the BNST, CeA, and pBLA regions (*n* = 3 mice per group). (D and F) Representative images of fluorescently labeled neurons in the BNST or CeA regions and the distribution of their axon terminals in the pBLA. Data are presented as mean ± SEM. AAV, adeno-associated virus; AP, anterior-posterior axis; DAPI, 4',6-diamidino-2-phenylindole; EGFP, enhanced green fluorescent protein; pBLA, posterior basolateral amygdala; aBLA, anterior basolateral amygdala; BNST, bed nucleus of the stria terminalis; CeA, central amygdala.


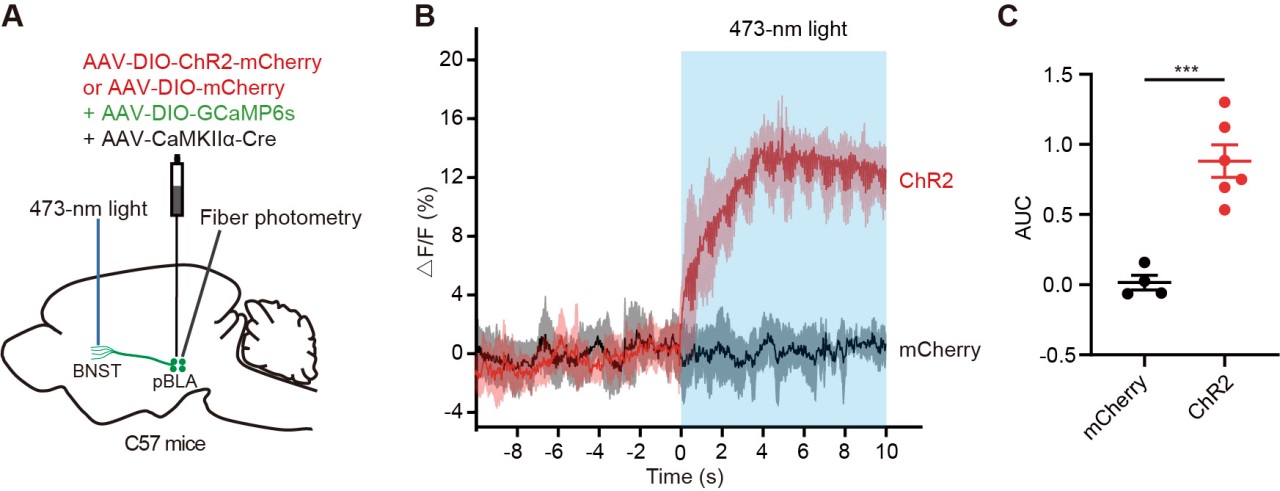


1. Figure S7. Optogenetic stimulation of pBLA glutamatergic axon terminals antidromically activates pBLA neuronal somas.

(A) Schematic of the strategy for optogenetically stimulating pBLA axon terminals while simultaneously monitoring pBLA neuronal soma activity. (B) Changes in calcium signals of pBLA glutamatergic neuronal somas during optogenetic stimulation of their axon terminals in the BNST. (mCherry: *n* = 4 mice; ChR2-mCherry: *n* = 6 mice). (C) Comparison of the area under the curve (AUC) between ChR2-mCherry and mCherry groups (mCherry: *n* = 4 mice; ChR2-mCherry: *n* = 6 mice; two-tailed unpaired t-test, ****P* < 0.001). Data are presented as mean ± SEM. AAV, adeno-associated virus; CaMKIIα, calcium/calmodulin-dependent protein kinase IIα; ChR2, channelrhodopsin-2; pBLA, posterior basolateral amygdala; BNST, bed nucleus of the stria terminalis.


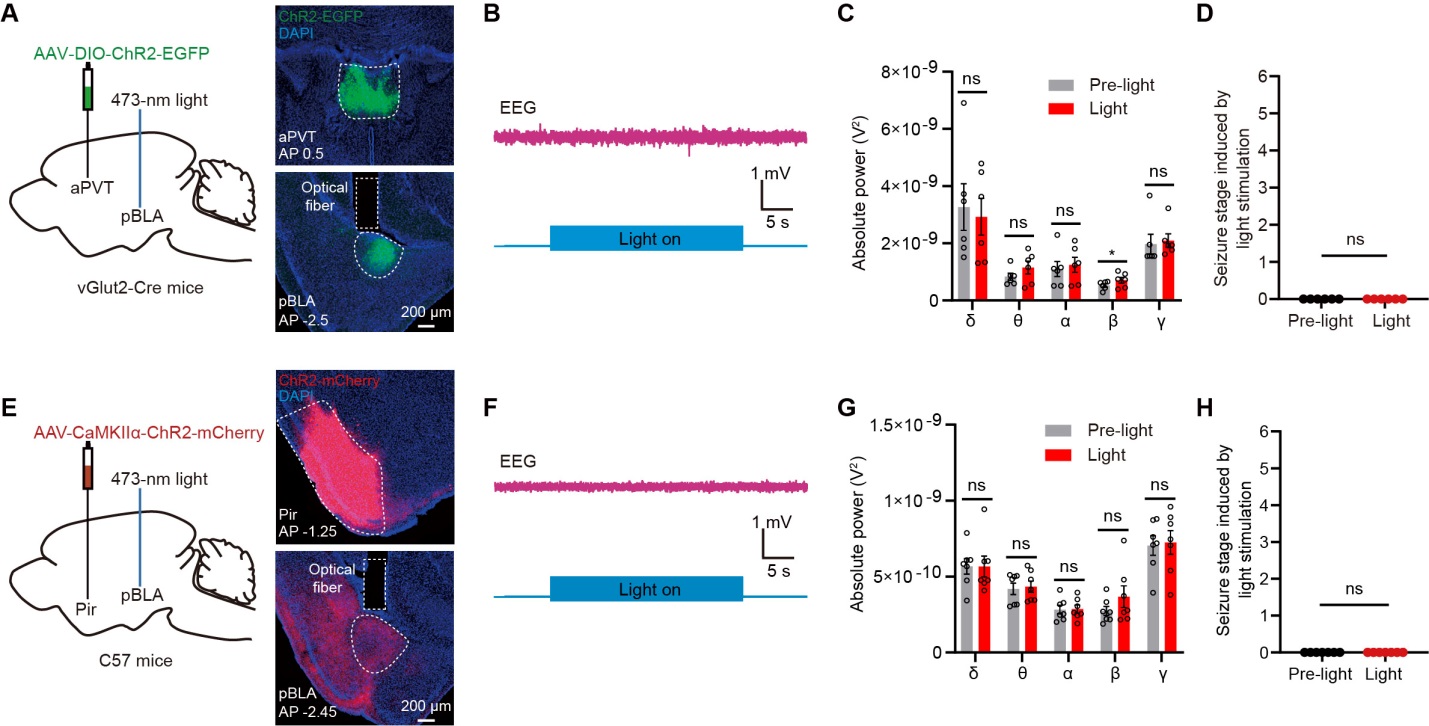


1. Figure S8. Optogenetic activation of the aPVT-pBLA or Pir-pBLA circuits does not result in epileptiform discharges or seizures.

(**A** and **E**) Left: Schematic of optogenetic activation of the aPVT-pBLA or Pir-pBLA circuits. Right: Virus expression in aPVT or Pir and the location of optical fibers above pBLA. (**B** and **F**) Representative EEG traces upon optogenetic activation of the aPVT-pBLA or Pir-pBLA circuits. (**C** and **G**) EEG spectral analysis before and after optogenetic activation of the aPVT-pBLA or Pir-pBLA circuits (aPVT-pBLA, *n* = 6 mice; Pir-pBLA, *n* = 7 mice; two-tailed paired t-test, ns, *P* > 0.05, **P* < 0.05). (**D** and **H**) Seizure stage upon optogenetic activation of the aPVT-pBLA or Pir-pBLA circuits (aPVT-pBLA, *n* = 6 mice; Pir-pBLA, *n* = 7 mice; Wilcoxon test, ns, *P* > 0.05). Data are presented as mean ± SEM. AAV, adeno-associated virus; AP, anterior-posterior axis; ChR2, channelrhodopsin-2; CaMKIIα, calcium/calmodulin-dependent protein kinase Iiα; DAPI, 4',6-diamidino-2-phenylindole; EEG, electroencephalography; EGFP, enhanced green fluorescent protein; aPVT, anterior paraventricular thalamus; Pir, piriform cortex; pBLA, posterior basolateral amygdala; vGluT2, vesicular glutamate transporter 2.


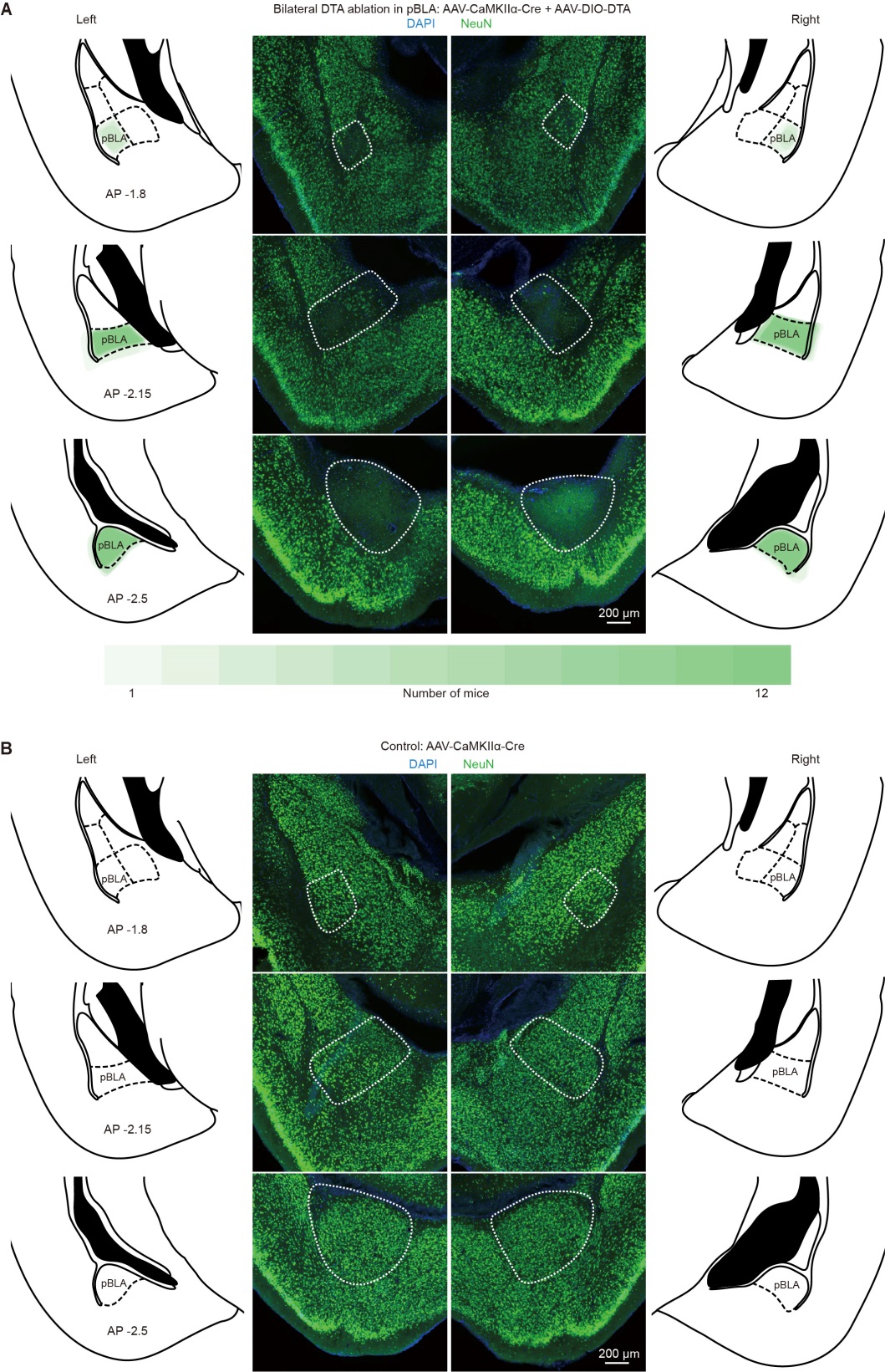


1. Figure S9. Bilateral DTA ablation in pBLA along the anterior-posterior axis.

(**A**) Representative images of NeuN (a marker protein for neuronal nuclei) staining for pBLA in DTA-injected mice along anterior-posterior axis and schematic diagram showing the overlay of DTA ablation areas in pBLA from 12 DTA group mice. (**B**) Representative images of NeuN staining for pBLA in control mice along anterior-posterior axis. AAV, adeno-associated virus; AP, anterior-posterior axis; CaMKIIα, calcium/calmodulin-dependent protein kinase IIα; DAPI, 4',6-diamidino-2-phenylindole; pBLA, posterior basolateral amygdala; DTA, diphtheria toxin A.


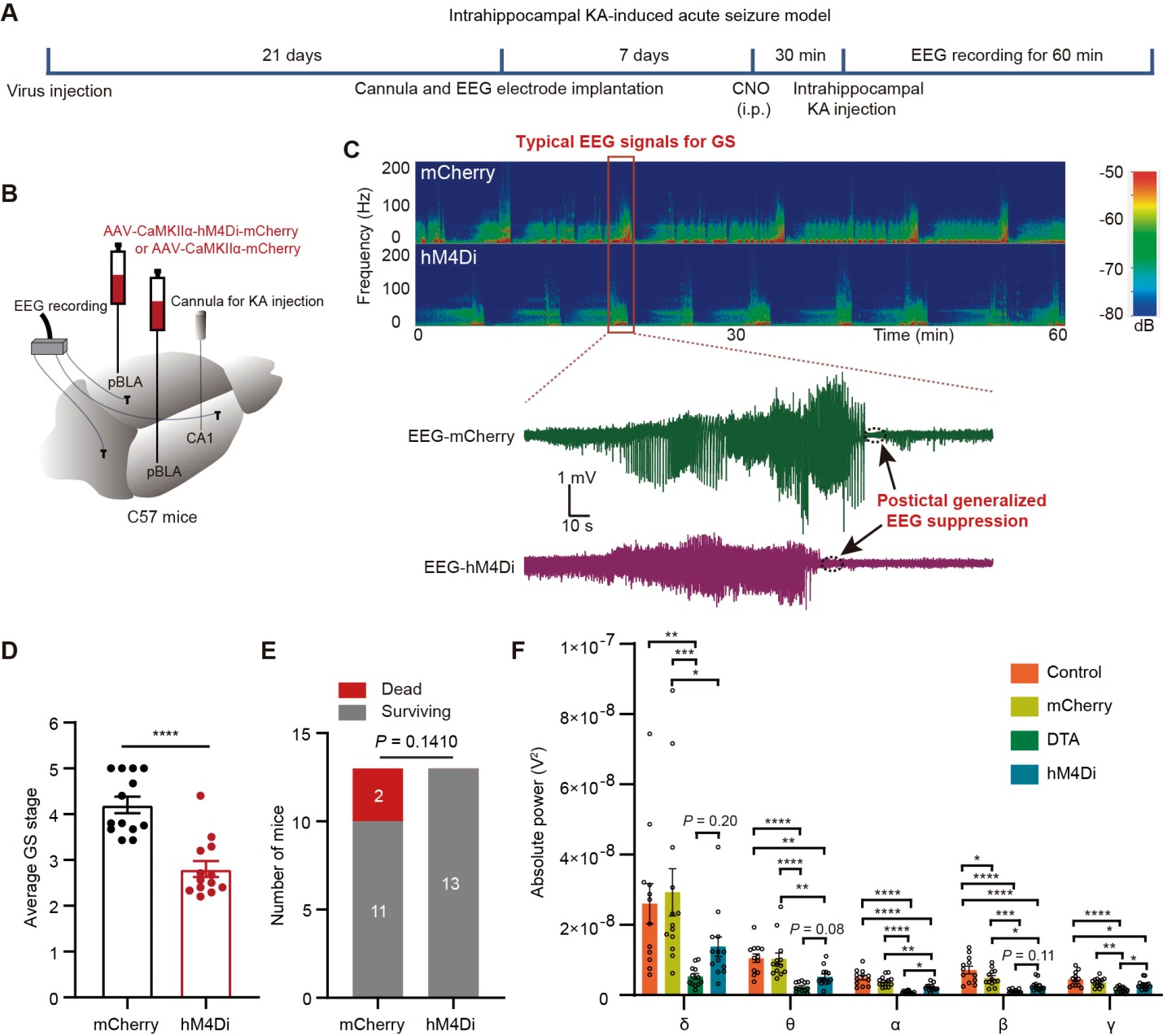


Figure S10. Chemogenetic inhibition of pBLA glutamatergic neurons significantly alleviates seizures in the intrahippocampal KA-induced acute seizure model.

(A) Schematic of the intrahippocampal KA-induced acute seizure model. (B) Schematic of EEG recordings. (C) Upper: Representative EEG power spectrum in acute seizure model. Lower: Enlarged view of typical EEG traces for GS from the red box in the upper panel. (D) Average GS stage within 60 minutes in the acute seizure model (*n* = 13 mice per group; Mann-Whitney U test, *****P <* 0.0001). (E) Number of dead and surviving mice in the acute seizure model (*n* = 13 mice per group; Chi-square test, *P* = 0.1410). (F) Spectral analysis of EEG signals for chemogenetic inhibition or DTA-mediated ablation of pBLA glutamatergic neurons ( data of Control and DTA groups from Figure 9F) in the acute seizure model (Control and DTA groups, *n* = 12 mice per group; mCherry and hM4Di groups, *n* = 13 mice per group; one-way ANOVA with Fisher’s least significant difference (LSD) test, **P <* 0.05, ***P <* 0.01, ****P* < 0.001, *****P* < 0.0001). Data are presented as mean ± SEM. AAV, adeno-associated virus; CaMKIIα, calcium/calmodulin-dependent protein kinase IIα; DTA, diphtheria toxin A; hM4Di, human muscarinic receptor 4; EEG, electroencephalography; KA, kainic acid; CNO, clozapine-N-oxide; i.p., intraperitoneal injection; pBLA, posterior basolateral amygdala; GS, generalized seizure.


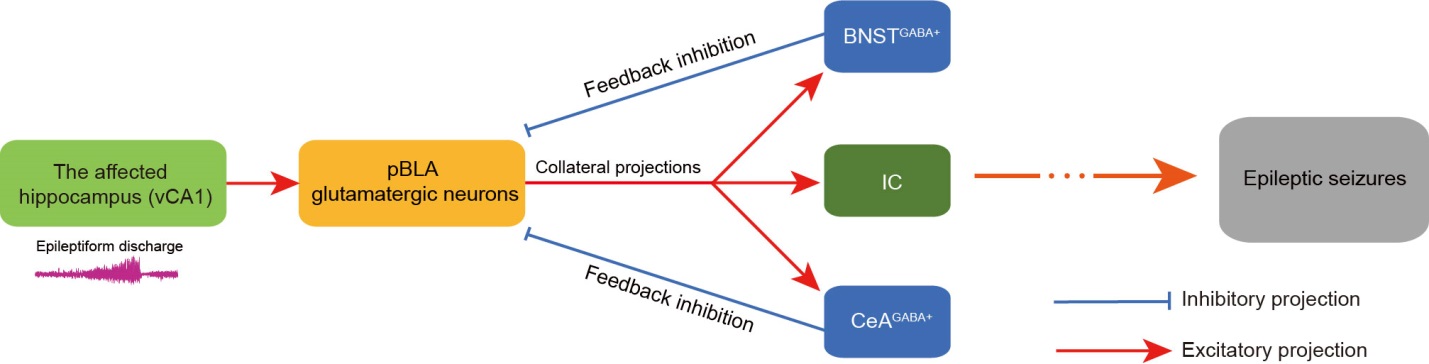


1. Figure S11. Model diagram showing the role of pBLA glutamatergic neurons and their collateral projections in epileptic seizures in TLE. Epileptiform discharges originating from the hippocampus spread to the pBLA in TLE mice. The pBLA glutamatergic neurons propagate these discharges via collateral projections to the IC, BNST, and CeA. The IC is a critical downstream region for the propagation of epileptiform discharges and seizure behaviors. In contrast, long-projecting GABAergic neurons in the BNST and CeA provide feedback inhibition to pBLA to balance the activity of glutamatergic neurons in the pBLA. vCA1, ventral hippocampal CA1; pBLA, posterior basolateral amygdala; IC, insular cortex; BNST, bed nucleus of the stria terminalis; CeA, central amygdala; TLE, temporal lobe epilepsy.
